# Evaluation of ecological suitability and quality of wild *Eleutherococcus senticosus* in Liaoning, China

**DOI:** 10.3389/fpls.2026.1849106

**Published:** 2026-06-30

**Authors:** Jiangli Xue, Xinyu Nie, Jiankui Zhang, Haibo Yin, Tingguo Kang

**Affiliations:** School of Pharmacy, Liaoning University of Traditional Chinese Medicine, Dalian, China

**Keywords:** ecological suitability, *Eleutherococcus senticosus*, geodetector, maximum entropy model, quality suitability

## Abstract

**Introduction:**

*Eleutherococcus senticosus*, a medicinal plant of significant value in China, currently faces habitat degradation and inconsistent quality. To establish a theoretical foundation for its effective conservation and utilization, this study takes Liaoning Province as the research area to investigate the distribution dynamics of this species under current and future climate scenarios, as well as the intrinsic mechanisms underlying habitat quality formation.

**Methods:**

This study integrated the Ultra-Performance Liquid Chromatography (UPLC), Maximum Entropy (MAXENT) and the coupled Maximum Entropy-Geographical Detector model (MAXENT-GD). Ecological Suitability and Quality of this species were analyzed under current and future climate scenarios. Model accuracy was evaluated using four validation metrics: coefficient of determination, ROC curve (AUC), Kappa coefficient, and True Skill Statistic (TSS).

**Results:**

Ten key environmental variables governed *E. senticosus* distribution: average precipitation in July (prec7), average precipitation in November (prec11), vegetation type (zblx), warmth index (index_ci), standard deviation of seasonal temperature variation (bio4), soiltype, average temperature in November (tmean11), aspect, altitude, and slope. Quality was primarily driven by interactive effects of precipitation, temperature, and topography. The overall accuracy, AUC value, Kappa coefficient, and TSS value of the MAXENT-GD model were 85.00%, 0.885, 0.700, and 0.7692, and the MAXENT model (82.00%, 0.862, 0.646, and 0.7231), indicating that the former possesses higher simulation accuracy. Under current climate conditions, the predicted suitable habitat area was 9.4129 × 10^4^ km² (MAXENT) and 7.1375 × 10^4^ km² (MAXENT-GD). Under future scenarios, the projected habitat change rates ranged from –22.14% to –26.52% (MAXENT) and –31.67% to –35.16% (MAXENT-GD), indicating a substantial threat from greenhouse gas emissions to habitat suitability.

**Discussion:**

We have established a comprehensive research framework capable of predicting both current and future ecological suitability and quality distribution for it. Furthermore, this study employed multiple validation methods to assist in model accuracy assessment, thereby enhancing the reliability of the results, with the aim of providing a reference for related research on other species and in other regions. Collectively, this approach provides a scientific basis for the introduction, domestication, and standardized cultivation of *E. senticosus*, thereby supporting the science-based planning and sustainable development of high-quality medicinal material production bases.

## 1 Introduction

Will climate change and habitat fragmentation lead to continued shrinkage of suitable habitats for the medicinal plant *Eleutherococcus senticosus* in Liaoning Province, thereby threatening the survival of its wild populations? As a national key protected medicinal species (Grade III) in China (National Key Protected Wild Medicinal Species List) and is classified as Endangered (EN) on the IUCN Red List (Species assessment and conservation), *E. senticosus* carries multiple values spanning clinical medicine, the broader health industry, and the culture of medicinal and edible homology (Chunmiao et al., 2023; Shulai et al., 2025; Ziying et al., 2025). Liaoning Province represents a critical region for the conservation and sustainable use of *E. senticosus* resources, yet it faces a pronounced contradiction between declining wild resources and growing market demand. To address this imbalance without competing for farmland, there is an urgent need to adopt cultivation strategies such as artificial regeneration, wild tending, and simulated wild cultivation. Successful implementation of these measures depends on a precise understanding of the ecological suitability of *E. senticosus* in Liaoning.

Although species distribution models have been widely applied to assess habitat suitability of medicinal plants, existing studies on *E. senticosus* suffer from several limitations: existing research specifically targeting this species remains relatively scarce; most available work has focused on broad-scale descriptions of suitable distribution areas (Ran et al., 2018), with no systematic spatial assessment conducted for Liaoning Province. the quantitative relationships between environmental variables and species distribution, along with their ecological explanatory power, remain insufficiently elucidated; and no studies have yet addressed the prediction of distribution patterns under future climate scenarios. These deficiencies render current conservation planning without precise spatial decision-making foundations, thereby limiting its effectiveness, and directly constrain the cognitive advancement from distribution prediction to driving mechanism interpretation. Therefore, there is an urgent need to conduct habitat suitability research for *E. senticosus* in Liaoning Province.

*Eleutherococcus senticosus* (Rupr. & Maxim.) Maxim. [Acanthopanax senticosus (Rupr. & Maxim.) Harms] is a perennial deciduous shrub of the family Araliaceae. Its roots, rhizomes, or stems are used medicinally (Pharmacopoeia of the People’s Republic of China (2025 Edition)) and exhibit multiple pharmacological activities, including antitumor, anti−fatigue, anti−inflammatory, antimicrobial, sedative, antihypertensive, and cardiocerebrovascular protective effects (Kos et al., 2025). The species is primarily distributed across Northeast and North China (Tianlei et al., 2024; Yijia et al., 2025; Xuchen and Dewen, n.d.) and is recognized as a genuine medicinal material (Daodi herb) of Liaoning Province (Zhiyao et al., 2025). In recent years, however, habitat alteration combined with overharvesting has caused a sharp decline in wild populations of *E. senticosus* in Liaoning (Chuancai and Minghua, 2021; Hongling et al., 2024). To formulate scientifically sound conservation measures and effectively support the development of cultivation techniques for sustainable resource use, it is particularly necessary to strengthen research on the resource distribution and ecological characteristics of *E. senticosus* in Liaoning Province.

In response to the above challenges, this study focuses on Liaoning Province as the study area. We introduce the MAXENT−GD (Maximum Entropy−Geographical Detector) model and validate model accuracy using independent field sample data, thereby enhancing the interpretability and reliability of predictions (Ying et al., 2016; Wang and Peng, 2022). Based on systematic field surveys and sample collection of *E. senticosus* resources in Liaoning, we use both the MAXENT and MAXENT−GD models to systematically assess ecological and quality−based habitat suitability patterns under current climate conditions and under three future climate scenarios (SSP1−2.6, SSP2−4.5, SSP5−8.5) for the 2050s (2041–2060) and 2090s (2081–2100). From the model outputs, we identify key environmental variables governing *E. senticosus* distribution, analyze their ecological threshold ranges, and reveal the species’ adaptive boundaries to climate change. The overarching aim is to provide a reference basis for the conservation and production planning of *E. senticosus* resources in Liaoning Province.

The specific objectives of this study are threefold: (1) to introduce the MAXENT−GD model to improve the interpretability and reliability of distribution predictions; (2) to predict the current distribution pattern of *E. senticosus* and the evolutionary trends of its suitable habitats under future climate scenarios; and (3) to analyze the key variables influencing *E. senticosus* growth, as well as the interactive and spatially heterogeneous effects of ecological drivers, thereby addressing the limited mechanistic interpretability of the standalone MAXENT model. It should be noted that the methods used here have certain limitations, and the conclusions require further validation and refinement.

## 2 Materials and methods

### 2.1 Data sources

#### 2.1.1 Species distribution data

**Figure 1:**
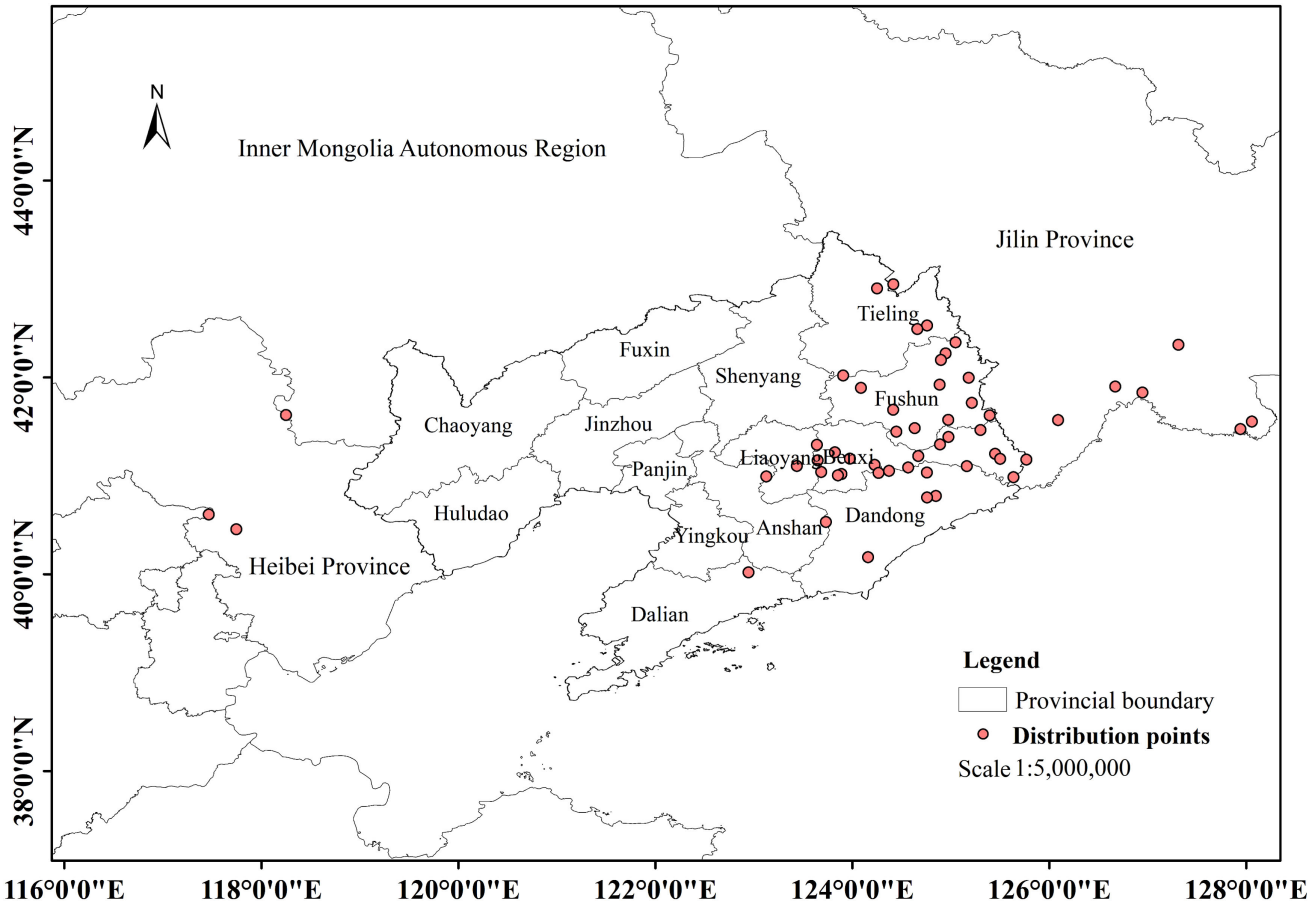
Distribution point of *E. senticosus* in Liaoning Province and surrounding areas.

There are three sources for the sampling point data of *E. senticosus*. (1) The field measurement data recorded from the end of June to the beginning of September 2025, recording the latitude and longitude information of the sampling points. (2) The Fourth Chinese (Liaoning Province) Traditional Chinese Medicine Resources Census Database, literature reports, and online databases. (3) Chinese Virtual Herbarium (CVH, https://www.cvh.ac.cn) and Global Biodiversity Information Facility (GBIF, https://www.gbif.org). Based on the above information, the geographical distribution data of *E. senticosus* were collected, recording the latitude and longitude as well as detailed distribution addresses, and the latitude and longitude information and specific locations were obtained and updated in the field using Global Positioning System (GPS) instruments. The original distribution records were screened: Geographic Information System software (ArcGIS) was used to perform buffer and analysis on the species distribution data, and 1 km was used as the threshold to remove duplicate records (Wu et al., 2025); records with coordinate precision lower than three decimal places (approximately 100 m) and low-precision points located in county centers were deleted; records labeled as “cultivated”, “medicinal garden”, “introduced”, and other non-wild records were excluded; further, a spatial thinning method was used, with 50 m as the minimum distance to reduce spatial autocorrelation (Remm and Remm, 2017). The Minimum Convex Polygon (MCP) of the retained records, buffered outward by 20 km (Jianya et al., 2015), was used as the accessible area (Barve et al., 2011). Finally, 54 effective sampling points of *E. senticosus* were obtained (**Figure 1**). All *E. senticosus* samples in this study were collected from Liaoning Province, a total of 47 batches. All medicinal materials were identified as the stems of *Eleutherococcus senticosus* (Rupr. ex Maxim.) Harms by Professor Zhang Jiankui of Liaoning University of Traditional Chinese Medicine. The sample diameters were 4 mm–6 mm. After natural drying in the shade, they were stored in the laboratory of Liaoning University of Traditional Chinese Medicine (**Supplementary Tables 1**, **2**).

#### 2.1.2 Environmental variables

Combining the characteristics of the suitable growth environment of *E. senticosus*, a total of 106 environmental Variables data including topography, soil, climate, precipitation, and vegetation type were selected for analysis. Among them, elevation data were sourced from the Geospatial Data Cloud Platform of the Chinese Academy of Sciences (https://www.gscloud.cn); topography, soil, integrated climate factors, and monthly average precipitation (January–February) data were sourced from the Traditional Chinese Medicine Resources Spatial Information Grid Database (http://www.tcm-resources.com); monthly average maximum temperature (January–December), monthly average minimum temperature (January–December), monthly average radiation intensity (January–December), and monthly average water vapor pressure (January–December) data were sourced from the WorldClim database (https://worldclim.org); monthly average temperature (January–December) and annual average temperature data were sourced from the National Tibetan Plateau Science Data Center (https://data.tpdc.ac.cn); vegetation type data were sourced from the Resource and Environment Science Data Center of the Chinese Academy of Sciences (https://www.resdc.cn). (**Supplementary Table 3**) Future climate data were sourced from the WorldClim database, using the sixth phase of the Coupled Model Intercomparison Project (CMIP6) (Tang et al., 2021), the second-generation Beijing Climate Center Climate System Model with medium resolution (BCC-CSM2-MR) suitable for China’s geographical environment (Su et al., 2020), and climate variable data under three scenarios of the Shared Socio-economic Pathways (SSPs) for the two periods of the 2050s and 2090s were downloaded from this model (Fick and Hijmans, 2017). The downloaded climate variables included: monthly average minimum temperature (January–December), monthly average maximum temperature (January–December), monthly total precipitation (January–December), and bioclimatic variables. The three scenarios included SSP1-2.6 (low forcing scenario), SSP2-4.5 (medium forcing scenario), and SSP5-8.5 (high forcing scenario) (Fan Zemeng, 2022). (**Supplementary Table 4**) The spatial resolution of all the above raster data (approximately 1.0 × 1.0 km) was 30 arc-seconds. To avoid the potential impact of multicollinearity on model stability (Gan et al., 2025), this study first used MAXENT 3.3.3 to exclude variables with zero contribution (Wu et al., 2025), then performed Spearman correlation analysis on the remaining environmental variables, retaining variables with |r|< 0.8. For variables with |r| ≥ 0.8, the variables that contributed significantly in the initial experiment were retained. (**Supplementary Table 5**) Finally, 10 variables were selected for modeling with the distribution points (He et al., 2026).

#### 2.1.3 Geographic information data

Map data were obtained from the DataV Data Visualization Platform (https://datav.aliyun.com/portal/school/atlas/areaselector?spm=a2c4g.11186623.0.0.e3d57e4coTNzVd) and downloaded as GeoJSON files. The data were converted using the mapshaper website and exported as Shapefile files.

### 2.2 Model development and evaluation

#### 2.2.1 Parameter settings

The MAXENT model is a probability model widely used in fields such as ecology, climate change, and biodiversity. Due to its good stability and high prediction accuracy, it has become one of the most commonly used methods in species distribution simulation research (Tang et al., 2021; Naqvi et al., 2026; Song et al., 2026; Zhang et al., 2026). In this paper, MAXENT 3.3.3 software was applied. The maximum number of iterations was set to 100,000, the convergence threshold was 0.0005, 15% of the distribution points were designated as the test set, and 85% were designated as the training set (Jiang et al., 2025). The number of repetitions was designated as 10, and the “bootstrap” iteration method was used (Zhao et al., 2025). The maximum number of background points was set to 10,000 (Barber Robert A. et al., 2022). Other parameters were the default values of the software. The model prediction results were saved as ASCII files. In the model, the response curve was used to evaluate the suitability range of Environmental variables, the area under the ROC curve (AUC) value was used to evaluate model accuracy, and the Jackknife test was used to examine the importance of Environmental variables.

#### 2.2.2 Model construction

The MAXENT model and the geographical detector were used together to construct the MAXENT-GD model. The prediction result ASCII file obtained from MAXENT was imported into ArcGIS 10.7 and converted into raster data. Through the ArcToolbox “Extract Multi Values to Points” function, the correlation data between environmental variables and ecological suitability of *E. senticosus* based on MAXENT were finally obtained. “Ecological density” (Ecology) was introduced as the dependent variable value of the original data, and the extracted values of each environmental variable were used as the independent variable values of the data (**Supplementary Table 6**). In R language version 4.4.3, the “GD” package (Song et al., 2020) and the optimal parameters geographical detector model (Chang’an and Naiju, 2025) were used to discretize the continuous variables, obtaining the optimal discretization method and optimal number of discretization intervals for each variable (Xin et al., 2023). In this paper, a total of five discretization methods were used, namely the quantile method, equal interval method, natural breaks method, geometric interval method, and standard deviation method (Mingyue et al., 2026; Yuan Yuan, 2026). factor detector, interaction detector, risk detector, and ecological detector analyses were carried out. Based on the q-value of each environmental Variable (from the factor detector), the degree of association between each environmental Variable and ecological density was determined (Guanghui et al., 2026), thereby revealing the influence of environmental variables on ecological suitability (Keyi et al., 2025). Environmental variables that had a significant influence on the dependent variable (based on the risk detector) were selected, and their q-values were normalized to obtain the optimized weight of each environmental variable. ArcGIS 10.7 was used for effective integration to obtain the ecological suitability model of *E. senticosus* based on the geographical detector, namely the MAXENT-GD coupled model (Equation 1) (Qi et al., 2026). The model construction for the ecological suitability of *E. senticosus* under future climate scenarios was performed similarly. The downloaded future climate data were transformed using ArcGIS 10.7, and MAXENT 3.3.3 was applied to obtain the ecological suitability model of *E. senticosus* under future climate conditions. The q-values of significantly influential Environmental variables were optimized for weighting to obtain the MAXENT model and the MAXENT-GD model for *E. senticosus* under three scenarios for the 2050s and 2090s.

(1) $$ESI_{MAXENT-GD}=\sum_{i=1}^{n}\left(\frac{q_{i}}{\sum_{j=1}^{n}q_{j}}\times Norm(X_{i})\right)$$

*ESI_MAXENT−GD_*: ecological suitability index optimized by the Geodetector

*q_i_*: explanatory power (q-value) of the i-th environmental factor from the Geodetector

*Norm(X_i_)*: normalized value (0–1) of the i-th environmental factor

#### 2.2.3 Model accuracy assessment

Four methods were applied in this study to validate model accuracy: the receiver operating characteristic (ROC) curve (BakhshiGanje et al., 2024), the matching coefficient between observed and predicted values, the kappa coefficient (Carpentier et al., 2017), and the True Skill Statistic (TSS) (Lazagabaster et al., 2024). The accuracy of the model results was evaluated using the area under the receiver operating characteristic curve (AUC-ROC), which ranges from 0 to 1. The closer the AUC value is to 1, the better the accuracy and reliability of the corresponding model (Ludwig et al., 2026). The Kappa coefficient ranges from -1 to 1, and its magnitude intuitively reflects the degree of agreement in classification results (Li et al., 2023; Martínez Pérez and Pérez Martin, 2023). TSS was used to evaluate the predictive performance of the species distribution model (Jagadesh et al., 2023; Xin et al., 2024). TSS ranges from -1 to 1, and values above 0.70 are considered indicative of models with excellent performance (Wu et al., 2025).

### 2.3 Methods for suitability analysis of *E. senticosus*

#### 2.3.1 Ecological suitability analysis of *E. senticosus*

Based on the results of the MAXENT and MAXENT-GD models, we used ArcGIS 10.7 software to visualize the output results, and applied the natural breaks classification method (Jenks) to divide the results into four suitability categories: poor suitable area (0.0–0.2), low suitable area (0.2–0.4), Medium suitable area (0.4–0.6), and high suitable area (0.6–1) (Chen et al., 2013; Li et al., 2024; Wang et al., 2026). Based on this classification principle, we delineated the ecologically suitable areas for *E. senticosus* under current and future climate change scenarios.

#### 2.3.2 Quality suitability analysis of *E. senticosus*

The following methods (Xue et al., 2016) were used to determine the content of active components in *E. senticosus*.

Instruments and reagents:

ACQUITY UPLC H-Class PLUS ultra-high performance liquid chromatograph (Agilent Technology Co., USA); KQ5200DB ultrasonic cleaner (Kunshan Ultrasonic Instruments Co., Ltd.); high-speed grinder for Chinese medicinal materials (Yongkang Fendou Industry and Trade Co., Zhejiang Province); JD60–4 electronic balance (Shenyang Longteng Electronics Co., Ltd.); CP225D electronic analytical balance (Sartorius, Germany); H/T16MM benchtop high-speed centrifuge (Hunan Hexi Instrumentation Equipment Co., Ltd.); Agilent ZORBAX Eclipse Plus C18 (3.0 × 100 mm, 1.8 μm) chromatographic column (Agilent Technology (China) Co., Ltd.). Six reference standards were used, namely syringin (batch number: B21684), 3,4-Dihydroxybenzoic acid (batch number: B21614), hyperoside (batch number: B20631), isofraxidin (batch number: B21547), chlorogenic acid (batch number: B20782), all purchased from Shanghai Yuanye Biotechnology Co., Ltd., and eleutheroside E (batch number: CFN99984) purchased from Wuhan Tianzhi Biotechnology Co., Ltd. The purity of the above reference standards was greater than 98%; purified water (Wahaha Co., Ltd.); formic acid (Shanghai Macklin Biochemical Technology Co., Ltd., chromatographic grade); methanol (Tianjin Kermiou Chemical Reagent Co., Ltd., analytical grade); ethanol (Tianjin Kermiou Chemical Reagent Co., Ltd., analytical grade); acetonitrile (Merck KGaA, chromatographic grade).

Standard and sample preparation:

An appropriate amount of reference standard powder was accurately weighed and dissolved in methanol solution (for eleutheroside E and hyperoside reference standards, 50% methanol was added first to dissolve) (Siyang et al., 2020) to prepare a mixed reference standard solution containing syringin, 3,4-Dihydroxybenzoic acid, eleutheroside E, hyperoside, isofraxidin, and chlorogenic acid at concentrations of 0.0600 mg·mL^-^¹, 0.1167 mg·mL^-^¹, 0.0400 mg·mL^-^¹, 0.0300 mg·mL^-^¹, 0.2400 mg·mL^-^¹, and 0.2000 mg·mL^-^¹, respectively. An appropriate amount of *E. senticosus* medicinal material was taken, crushed, and passed through a 40-mesh sieve. Approximately 2 g of powder was accurately weighed, transferred to a stoppered conical flask, and 24 mL of 80% methanol (Juan et al., 2024) was added. After sealing, the mass was weighed. Ultrasonic treatment (40 °C, 40 kHz, 300 W) was performed for 30 min, then cooled to room temperature, and the lost mass was supplemented with 80% methanol. After shaking well, centrifugation (Hexiang et al., 2024) (350 W, 50/60 Hz, 4000 r/min) was performed for 10 min. The supernatant was collected and passed through a 0.22 μm filter membrane, and the subsequent filtrate was collected to obtain the test solution.

Chromatographic conditions:

Chromatographic column: Agilent ZORBAX Eclipse Plus C18 (3.0 × 100 mm, 1.8 μm); mobile phase: 0.1% formic acid in water (A) – 0.1% formic acid in acetonitrile (B); gradient elution program: (0–6 min, 5% A; 6–8 min, 5%–8% A; 8–15 min, 8%–15% A; 15–20 min, 15%–26% A; 20–23 min, 26%–52% A; 23–25 min, 52%–100% A; 25–30 min, 100% A); detection wavelengths: 260 nm and 330 nm; flow rate: 0.25 mL·min^-^¹; column temperature: 40 °C; injection volume: 0.10 μL. Under these chromatographic conditions, the chromatographic peaks of each component were well separated.

Quality zoning methods:

On the basis of ecological suitability zoning, Statistical Package for the Social Sciences (SPSS 26.0) was used to perform Pearson correlation analysis (for data conforming to normal distribution) and Spearman correlation analysis (for data not conforming to normal distribution) between the active components (Pengcheng et al., 2025) and Environmental variables. Based on the correlation analysis results, Environmental variables that had a significant correlation (P< 0.05) with the six active components were selected. When the correlation coefficient between two environmental variables reached or exceeded 0.8, the variable with the higher contribution rate was retained. Finally, partial least squares (PLS) regression analysis (Bin et al., 2024) was performed to construct a relationship model between the chemical components of *E. senticosus* and the environmental variables. The raster calculator tool in ArcGIS 10.7 was used for calculation to obtain the single-index quality zoning for *E. senticosus*. The Analytic Hierarchy Process (AHP) was selected to perform pairwise comparisons of the six active components and to conduct a multi-active comprehensive evaluation of the active components. The consistency of the judgment matrix was tested. A CR< 0.1 indicated that the result was reasonable and consistent with the consistency requirement, and the weight coefficient of each active component was obtained. The multi-index comprehensive quality zoning result for *E. senticosus* was obtained through ArcGIS 10.7 (Miao et al., 2023).

## 3 Results

### 3.1 Ecological suitability analysis

#### 3.1.1 Key environmental variables and model evaluation

**Figure 2:**
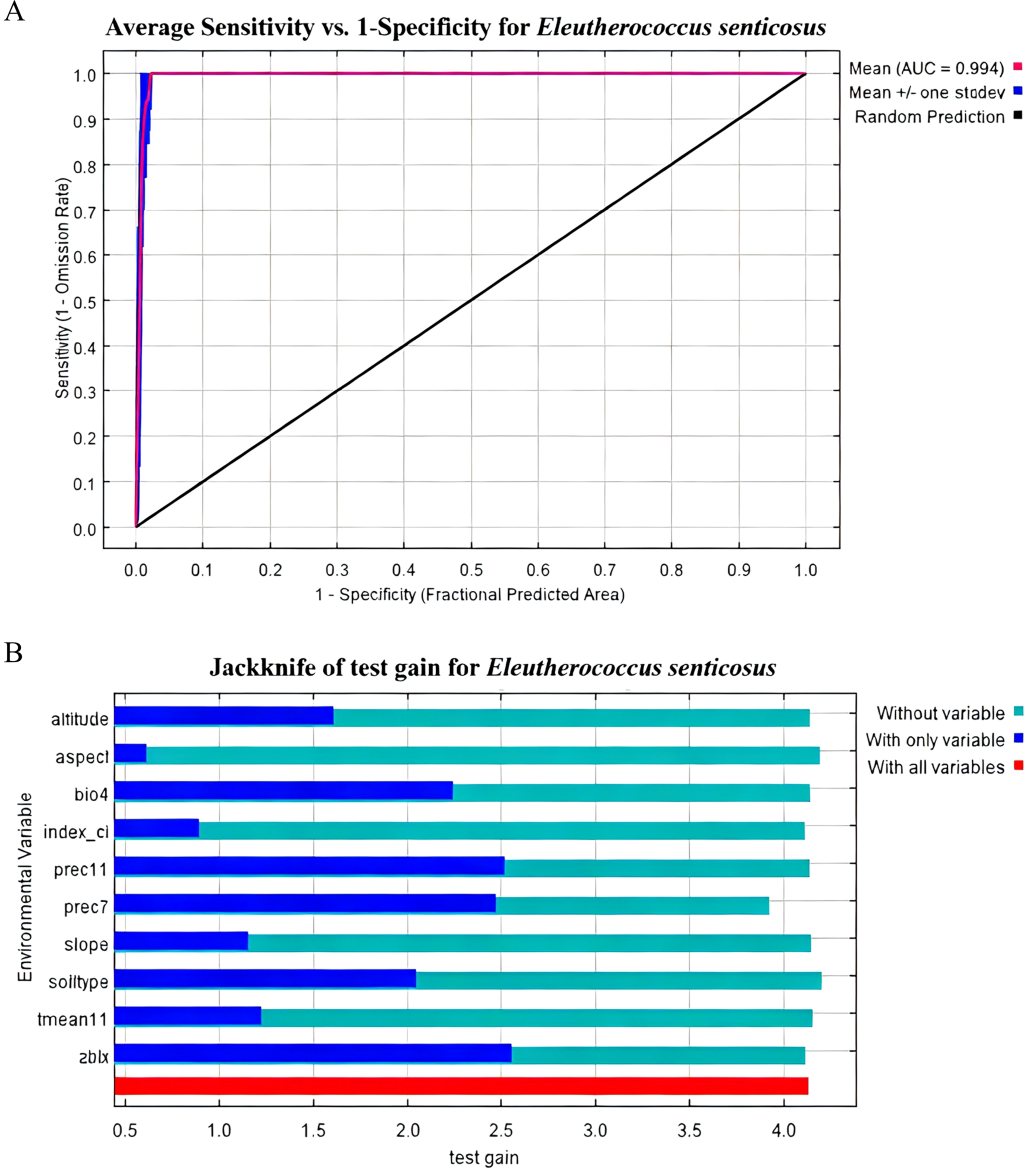
Model accuracy evaluation and the importance of the variables for MAXENT. **(A)** ROC analysis of MAXENT model for predicting the distribution of *E. senticosus*. **(B)** Jackknife test results for environmental variables on *E. senticosus*.

**Figure 3:**
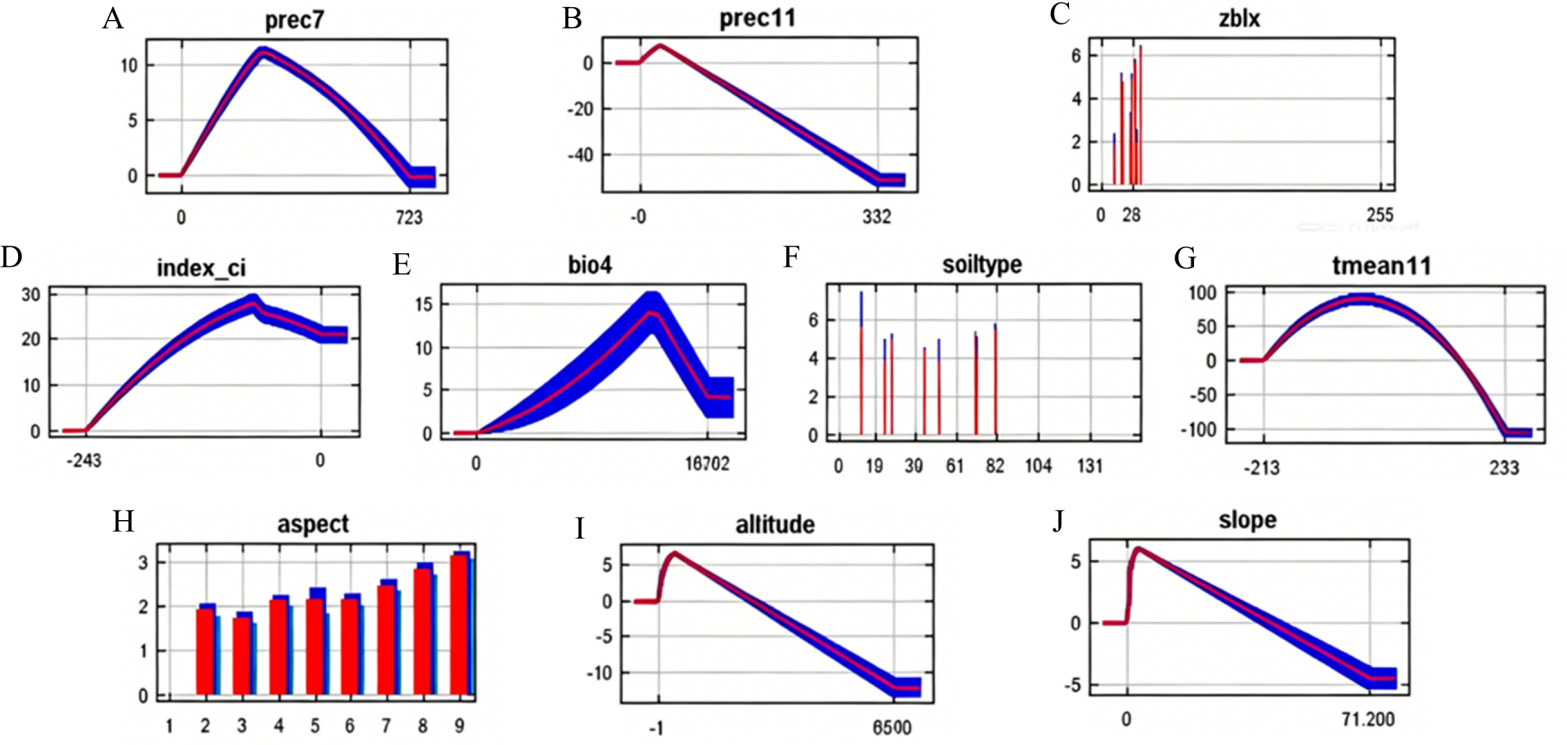
Response curve of *E. senticosus* existence probability to the dominant environmental variables: **(A)** Average precipitation in July. **(B)** Average precipitation in November. **(C)** Vegetation type **(D)** Warmth index **(E)** Standard deviation of seasonal temperature variation **(F)** Soiltype **(G)** Average temperature in November **(H)** Aspect **(I)** Altitude **(J)** Slope.

Through the calculation of the MAXENT model, under the current default parameter settings, the 10th percentile test omission rate of the model was 12.5%, and the balance threshold test omission rate was 0%, indicating that the model’s predictive ability for the test samples was excellent, and no actual distribution point was incorrectly classified as a non-suitable area (Nan et al., 2020). The average AUC of the ROC curves of the 10 runs was 0.994 (>0.9), indicating that the model accuracy was high and the prediction results were reliable (Zhang et al., 2024) and it could be used to predict the ecological suitability distribution of *E. senticosus* in Liaoning Province (**Figure 2A**). The Jackknife program examined the importance of each environmental variable (**Figure 2B**). A total of 10 key environmental variables with non-zero contribution rates were screened out, namely prec7, prec11, zblx, index_ci, bio4, soiltype, tmean11, aspect, altitude, and slope (**Table 1**). The optimal range of key environmental variables was defined by the probability of occurrence of this species greater than 0.5 (**Figure 3**) (Ren et al., 2025). The results showed that precipitation, temperature, and geographical factors were the main environmental variables affecting the growth and distribution of *E. senticosus*. Finally, combined with the contribution rate and correlation analysis, these 10 environmental variables were further analyzed.The results of using four methods for accuracy validation and model comparison analysis are as follows:

**Table 1:** Key environmental Variables for *E.* identified by MAXENT model.

Environmental variables	Description	Contribution rate (%)	Suitable range
prec7	Mean precipitation in July	31.9	493~795mm
prec11	Mean precipitation in November	19.1	23~34mm
zblx	Vegetation type	15.9	Temperate coniferous and deciduous broad-leaved mixed forest
index_ci	Warmth index	14.1	-82~-66
bio4	Temperature seasonality	8.7	12~13°C
soiltype	Soil subgroup	8.4	Humic Cambisols (CMu)
tmean11	Mean temperature in November	0.8	-50~-15°C
aspect	Aspect	0.7	Northwest, 292.5-337.5°
altitude	Elevation	0.5	310~671m
slope	Elevation	0.1	2~4°

1. Validation of matching coefficient between true values and predicted values

Based on the field-collected sample points and pseudo-absence distribution point data, the ecological suitability results were validated. First, the target survey area was delineated. Vegetation type, rivers, human settlements, and cultivation bases were used as screening conditions to exclude obviously unsuitable and non-wild areas. Then, a buffer distance of 5 km was set centered on each sampling point (Huilin et al., 2022). Within the area outside the buffer zone but within the target survey area, 100 pseudo-absence points were randomly generated (Senay et al., 2017; Hongzhao et al., 2026). On this basis, a fishnet grid layer was created to construct binary classification variables of presence (assigned value 1) and pseudo-absence (assigned value 0). To evaluate changes in the scope, the continuous suitability mapping was converted into a binary layer by applying a minimum suitability threshold of 0.2 (corresponding to the minimum value of the low suitability category) (Zhuo et al., 2024). Subsequently, SPSS 26.0 was used to construct a presence-absence confusion matrix (**Table 2**), and the matching accuracy between the true values and the predicted values was calculated. The results showed that the prediction accuracy of the MAXENT model for pseudo-absence and presence was 100.00% and 66.04%, respectively, with an overall accuracy of 82.00%; whereas the MAXENT-GD model showed prediction accuracy of 100.00% for pseudo-absence and 70.00% for presence, with the overall accuracy increasing to 85.00%, demonstrating superior predictive performance.

**Table 2:** Confusion matrix.

Model	Class	Predicted Presence	Predicted Absence	Accuracy (%)	Overall accuracy
MAXENT	Actual Presence	47	18	100.00%	82.00%
	Actual Absence	0	35	66.04%	
MAXENT-GD	Actual Presence	50	15	100.00%	85.00%
	Actual Absence	0	35	70.00%	

2. ROC curve validation

ROC curve validation using a different algorithm from the MAXENT model was performed: the area under the ROC curve (AUC value) was used as an effective evaluation of model accuracy. The results showed that the AUC value of the MAXENT model was 0.862, and the AUC value of the MAXENT-GD model was 0.885. The latter had a larger AUC value, indicating its higher prediction accuracy and better result reliability.

3. Kappa coefficient validation

The Kappa coefficient was calculated based on the confusion matrix. The Kappa coefficient of the MAXENT model was 0.646; the Kappa coefficient of the MAXENT-GD model was 0.700. The latter reached a high level of agreement, indicating that its prediction results had higher consistency with the actual situation.

4. TSS validation

The TSS value of the MAXENT model was 0.7231; the TSS value of the MAXENT-GD model was 0.7692. The latter was significantly higher than the former, indicating that the MAXENT-GD model had better predictive ability.

Based on the above four validation methods, the MAXENT-GD model outperformed the MAXENT model in terms of the matching coefficient between true values and predicted values, ROC curve, Kappa agreement, and TSS value, demonstrating higher prediction accuracy and better fitting ability to the actual distribution of *E. senticosus*. Therefore, the results analysis of this study will focus on the output of the MAXENT-GD model.

#### 3.1.2 Ecological suitability zoning results and their distribution areas

**Figure 4:**
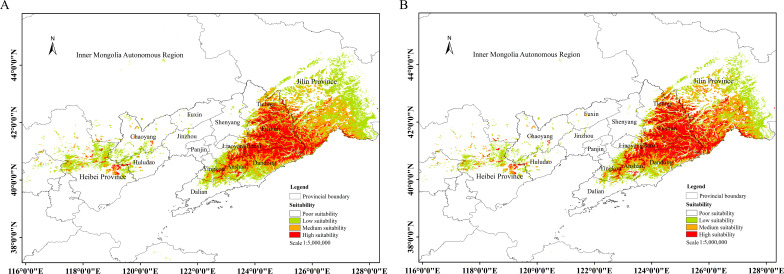
Predicted distribution of *E. senticosus* in Liaoning Province under current climate conditions. **(A)** Predicted distribution based on the MAXENT model. **(B)** Predicted distribution based on the MAXENT-GD coupled model.

Based on the prediction results of the current ecological suitability distribution of *E. senticosus* from the MAXENT model and the MAXENT-GD model (**Figure 4**), this study used ArcGIS software to reclassify the model outputs and calculate the area of each suitability class. The results showed that: under the MAXENT model, the areas of thehigh suitable area, Medium suitable area, low suitable area, for wild *E. senticosus* in Liaoning Province were 3.3169 × 10^4^ km², 2.5278 × 10^4^ km², and 3.5682 × 10^4^ km², respectively, with a total suitable area of 9.4129 × 10^4^ km², accounting for 63.30% of the total area of Liaoning Province. Under the MAXENT-GD model, the areas of high suitable area Medium suitable area and low suitable area were 2.6945 × 10^4^ km², 2.0796 × 10^4^ km², and 2.3634 × 10^4^ km², respectively, with a total suitable area of 7.1375 × 10^4^ km², accounting for 48.00% of the total area of Liaoning Province. The prediction results of the two models were generally consistent, both showing that the ecological suitability zones of *E. senticosus* are mainly distributed in the eastern mountainous areas of Liaoning Province, covering the entire territory of Fushun City and Benxi City, as well as eastern Tieling City, northern Dandong City, southeastern Anshan City, and southeastern Yingkou City, which were generally consistent with the existing recorded distribution records of *E. senticosus*. It is worth mentioning that, in order to better connect and transition with the zoning of *E. senticosus* in neighboring provinces, we retained the zoning results for areas of neighboring provinces adjacent to Liaoning Province (Xiaowu and Xibao, 2022).

#### 3.1.3 Results of spatial differentiation from the Geodetector

The research results showed that the optimal discretization methods for different Environmental variables varied: prec7, zblx, and soiltype were most suitable for the natural breaks classification method; prec11, bio4, tmean11, and aspect were most suitable for the standard deviation classification method; index_ci and altitude were most suitable for the equal interval classification method; and slope was most suitable for the geometric interval classification method. After classification by their respective optimal discretization methods, the q-values of all Environmental variables were significantly better than those of other methods. Meanwhile, the optimal numbers of discretization intervals were: zblx, bio4, and soiltype had 4 intervals, and the remaining Environmental variables had 5 intervals. The combination of spatial discretization method and number of intervals had a significant impact on the q-value.

1. Results of the factor detector

The explanatory power (q-value) of each environmental variable on the ecological suitability of *E. senticosus* was as follows: prec7 (0.5687), prec11 (0.4986), bio4 (0.4268), altitude (0.3676), tmean11 (0.3638), index_ci (0.3061), soiltype (0.1856), aspect (0.1164), slope (0.1054), zblx (0.1025). Among them, prec7 had the highest explanatory power (0.5687), and slope had the lowest explanatory power (0.1054). The significance test results showed that, except for zblx, soiltype, aspect, and slope, the remaining environmental variables all exhibited significant differences (p< 0.05), indicating that these variables have a significant impact on the ecological suitability of *E. senticosus*. Although the above four variables did not reach a significant level, they still have certain statistical significance.

2. Results of the interaction detector

The analysis results of the interaction detector showed that when any two environmental variables acted together, the impact on the ecological suitability of *E. senticosus* presented two scenarios: enhancement or weakening. Among them, the interaction between prec7 and altitude had the greatest impact, showing bifactorial enhancement, with a q-value of 0.7445; while the interaction between altitude and slope had the smallest impact, showing unifactorial nonlinear weakening, with a q-value of 0.12. Overall, the majority of groups showed enhancement under the interaction of two environmental variables, while fewer groups showed weakening, and no case occurred where the interaction effect was equal to the effect of any single factor alone.

3. Results of the ecological detector

The ecological detector was used to compare whether there is a significant difference in the impact of any two independent variables on the spatial distribution of the dependent variable, that is, to determine whether the influence of each pair of environmental variables on the ecological suitability of *E. senticosus* is statistically significant. According to the detection results, “Y” (Yes) indicates that there is a significant difference between two environmental variables, meaning that their impacts on the ecological suitability of *E. senticosus* are significantly different; “N” (No) indicates that there is no significant difference between the two, meaning that the degrees of impact are comparable. The results are shown in the figure.

4. Results of the risk detector

The risk detector determines whether there are significant differences among different partitions within an independent variable itself. The results showed that for the three environmental variables zblx, aspect, and slope, there were no significant differences among their respective partitions, while for the remaining environmental variables, there were significant differences among their partitions.The risk detector is used to determine whether there are significant differences among different partitions within the same environmental variables. The results showed that for the three environmental variables zblx, aspect, and slope, there were no significant differences among their respective partitions, while for the remaining environmental variables, there were significant differences among their partitions (**Supplementary Figures 1**-**6**).

In summary, precipitation, temperature, and altitude are the key environmental variables controlling the ecological suitability distribution of wild *E. senticosus* in Liaoning Province. At the same time, the interactions among environmental variables cannot be ignored. Relevant conservation and cultivation decisions should prioritize the development of zone-specific management strategies based on the above key variables and their interactions, in order to achieve the sustainable utilization and scientific management of *E. senticosus* resources, and thereby attain the synergistic goals of resource conservation and industrial development.

#### 3.1.4 Future ecological suitability distribution results for *E. senticosus*

**Figure 5:**
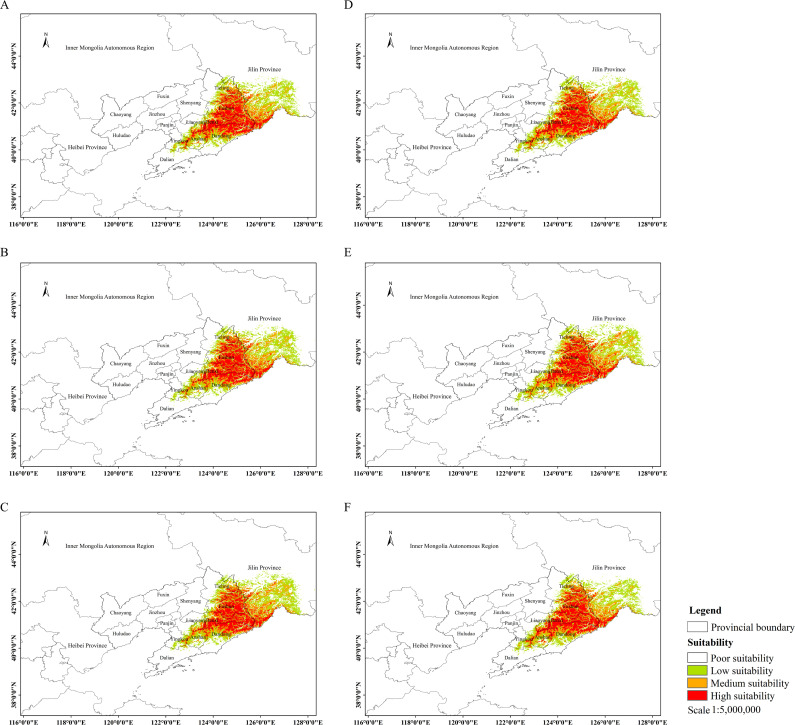
Predicted distribution of *E. senticosus* in Liaoning Province under future climate conditions. Six future climate scenarios predicted based on the MAXENT model. **(A)** Distribution under SSP1-2.6 in the 2050s. **(B)** Distribution under SSP2-4.5 in the 2050s. **(C)** Distribution under SSP5-8.5 in the 2050s. **(D)** Distribution under SSP1-2.6 in the 2090s. **(E)** Distribution under SSP2-4.5 in the 2090s. **(F)** Distribution under SSP5-8.5 in the 2090s.

**Figure 6:**
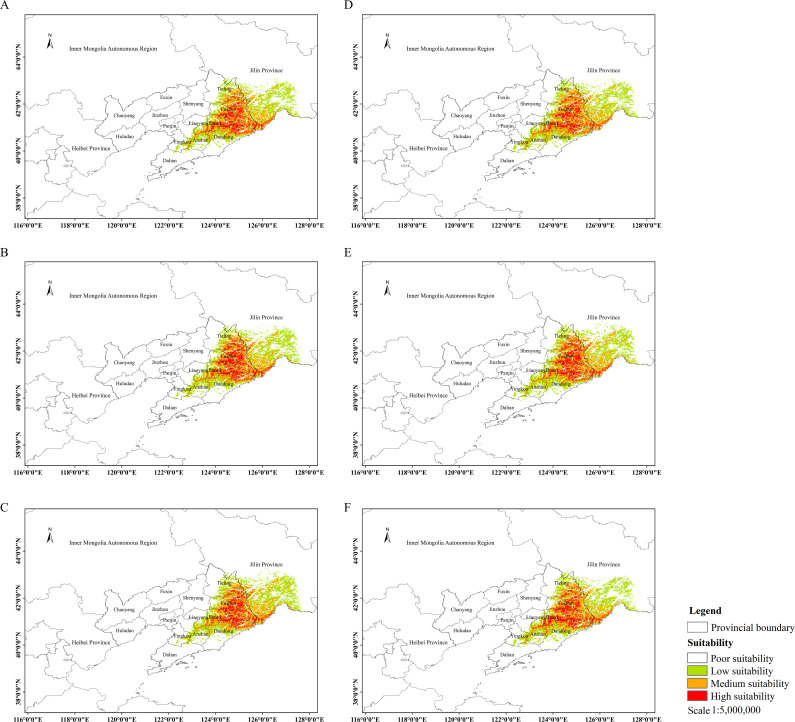
Predicted distribution of *E. senticosus* in Liaoning Province under future climate conditions. Six future climate scenarios predicted based on the MAXENT-GD coupled model. **(A)** Distribution under SSP1-2.6 in the 2050s. **(B)** Distribution under SSP2-4.5 in the 2050s. **(C)** Distribution under SSP5-8.5 in the 2050s. **(D)** Distribution under SSP1-2.6 in the 2090s. **(E)** Distribution under SSP2-4.5 in the 2090s. **(F)** Distribution under SSP5-8.5 in the 2090s.

Under the background of climate change, this study selected two climate scenarios (the 2050s and the 2090s) and used the MAXENT model and the MAXENT-GD model respectively to predict the potential distribution areas of future ecological suitability of *E. senticosus* (**Figures 5**, **6**). To quantify the distribution changes of suitable areas for *E. senticosus* under current and future climate conditions, the Suitable Habitat Change Rate (SHCR) was introduced for evaluation. The prediction results showed that under the framework of both the MAXENT model and the MAXENT-GD model, the suitable area for *E. senticosus* showed a decreasing trend across the six climate scenarios of the 2050s and 2090s. Compared with current climate conditions, the total suitable area under future scenarios decreased by 22.14%–26.52% and 31.67%–35.16%, respectively (**Table 3**). The changing trends of the total suitable area for *E. senticosus* predicted by the two models were generally consistent. Specifically, in the 2050s, the total suitable area showed a decreasing trend with increasing carbon emission concentration; whereas in the 2090s, the total suitable area showed an increasing trend with increasing carbon emission concentration. Within the same time period, the total suitable area changed significantly under different emission intensity scenarios, indicating that CO_2_ emission concentration has a substantial impact on the suitable area for *E. senticosus*. The above results reveal the high sensitivity of the ecological suitability of *E. senticosus* to the magnitude of changes in climatic factors. Accordingly, when formulating relevant conservation and cultivation strategies, one should not merely rely on the qualitative judgment of whether the climate shows a warming trend, but should further pay attention to quantitative dimensions that characterize differences in emission intensity, such as the rate of temperature increase and the magnitude of temperature rise.

**Table 3:** Results of distribution area of *E. senticosus*.

Model	Scenario	Area(×10^4^km^2^)				Change rate of suitable habitat			
		L	M	H	T	L	M	H	T
MAXENT	ssp126-2050s	2.66	2.07	2.40	7.12	-25.58%	-18.14%	-27.75%	-24.35%
	ssp245-2050s	2.61	1.96	2.48	7.05	-26.91%	-22.59%	-25.18%	-25.14%
	ssp585-2050s	2.60	1.99	2.33	6.92	-27.12%	-21.44%	-29.74%	-26.52%
	ssp126-2090s	2.58	2.11	2.46	7.15	-27.61%	-16.61%	-25.96%	-24.07%
	ssp245-2090s	2.66	2.09	2.52	7.27	-25.53%	-17.15%	-24.08%	-22.77%
	ssp585-2090s	2.64	2.12	2.57	7.33	-26.00%	-16.22%	-22.50%	-22.14%
		L	M	H	T	L	M	H	T
MAXENT-GD	ssp126-2050s	2.29	1.85	2.15	6.28	-3.06%	-11.21%	-20.33%	-33.24%
	ssp245-2050s	2.26	1.76	2.19	6.20	-4.57%	-15.53%	-18.85%	-34.15%
	ssp585-2050s	2.29	1.77	2.04	6.10	-2.99%	-14.89%	-24.27%	-35.16%
	ssp126-2090s	2.27	1.85	2.21	6.33	-3.82%	-11.00%	-18.14%	-32.76%
	ssp245-2090s	2.33	1.87	2.16	6.36	-1.39%	-10.04%	-19.77%	-32.40%
	ssp585-2090s	2.25	1.91	2.28	6.43	-4.86%	-8.37%	-15.45%	-31.67%

L, Low; M, Medium; H, High; T, Total.

Under future climate change scenarios, the habitat of *E. senticosus* in Liaoning Province shows a certain degree of degradation trend, with the area of each suitability class decreasing. Among them, Fushun City, Benxi City, eastern Tieling City, and northern Dandong City remain the high suitable planting areas, and these areas can serve as key development zones for simulating wild cultivation of *E. senticosus* in Liaoning Province over the next half century. It should be noted that simulating wild cultivation should adhere to the principle of adapting measures to local conditions. In addition, when artificially introducing the species to new suitable areas, attention should be paid to ecological environmental protection, the planting layout should be rationally planned, the planting scale should be strictly controlled, and native vegetation should be preserved, so as to minimize potential interference with the structure of local plant communities and interspecific competitive relationships, thereby maintaining the integrity and stability of the ecosystem.

### 3.2 Quality suitability analysis

#### 3.2.1 Component content determination and correlation analysis results between components and environmental variables

The results of the component analysis of *E. senticosus* are as follows (details are shown in **Supplementary Table 7** of the **Supplementary Materials**). The linear correlation coefficients (r) for syringin, 3,4-Dihydroxybenzoic acid, eleutheroside E, hyperoside, isofraxidin, and chlorogenic acid were 0.9996, 0.9999, 0.9996, 0.9998, 0.9999, and 0.9996, respectively (**Supplementary Table 8**). The relative standard deviations (RSD) for precision were 0.96%, 1.58%, 0.99%, 0.84%, 0.62%, and 0.86%, respectively; the RSDs for repeatability were 0.97%, 1.29%, 1.75%, 2.52%, 2.29%, and 2.09%, respectively; the RSDs for stability were 1.47%, 2.32%, 0.62%, 2.23%, 2.16%, and 0.92%, respectively; the average recoveries were 99.56%, 100.28%, 98.37%, 99.63%, 99.20%, and 98.54%, with corresponding RSDs of 1.87%, 1.35%, 1.21%, 1.85%, 1.96%, and 1.04%, respectively. On this basis, we performed Spearman correlation analysis between the six active components and the 10 environmental variables, and screened out a total of 6 environmental variables that showed significant correlation with the chemical components (**Supplementary Table 9**) for subsequent construction of regression equations (**Supplementary Table 10**). Partial least squares (PLS) regression analysis (multivariate) and linear regression analysis (univariate) were used to construct single-index quality zoning models. Furthermore, the Analytic Hierarchy Process (AHP) was used to construct a judgment matrix (**Supplementary Table 11**), and the effective weight of each component was determined through the consistency test results (CR< 0.1) (**Supplementary Table 12**). Finally, the raster calculator was used to perform raster calculation and spatial zoning of comprehensive quality.

#### 3.2.2 Quality suitability analysis results of *E. senticosus*

**Figure 7:**
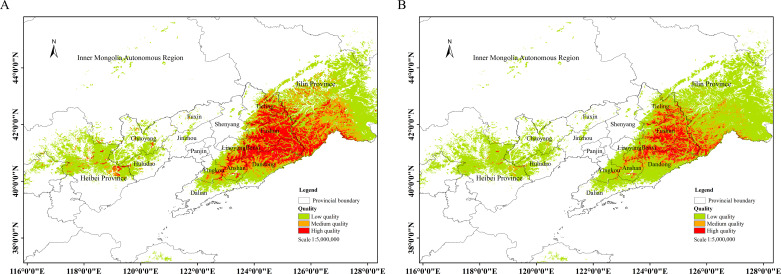
Predicted quality of *E. senticosus* in Liaoning Province under current climate conditions. **(A)** Predicted quality based on the MAXENT model. **(B)** Predicted quality based on the MAXENT-GD coupled model.

**Figure 8:**
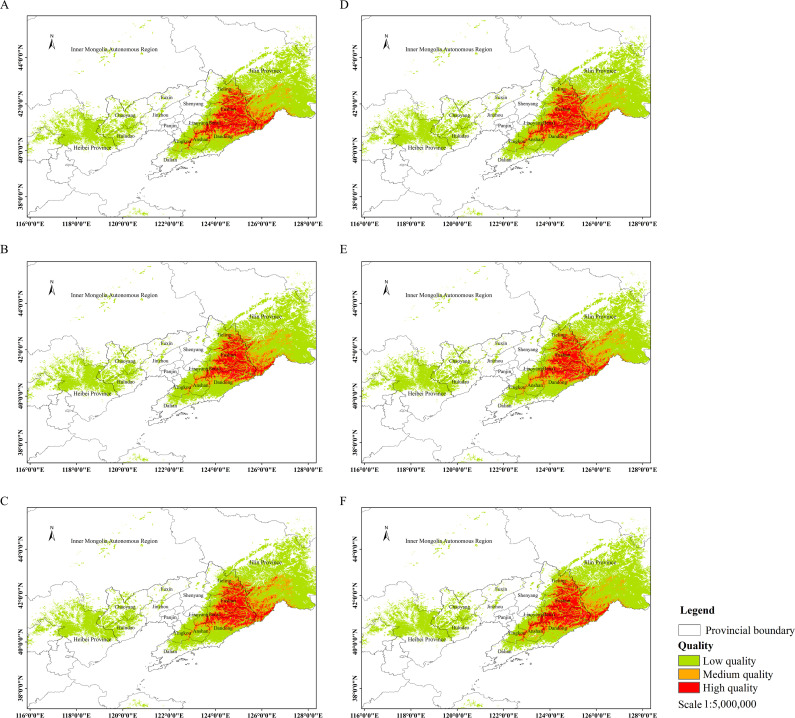
Predicted quality of *E. senticosus* in Liaoning Province under future climate conditions. Six future climate scenarios predicted based on the MAXENT model. **(A)** Quality under SSP1-2.6 in the 2050s. **(B)** Quality under SSP2-4.5 in the 2050s. **(C)** Quality under SSP5-8.5 in the 2050s. **(D)** Quality under SSP1-2.6 in the 2090s. **(E)** Quality under SSP2-4.5 in the 2090s. **(F)** Quality under SSP5-8.5 in the 2090s.

**Figure 9:**
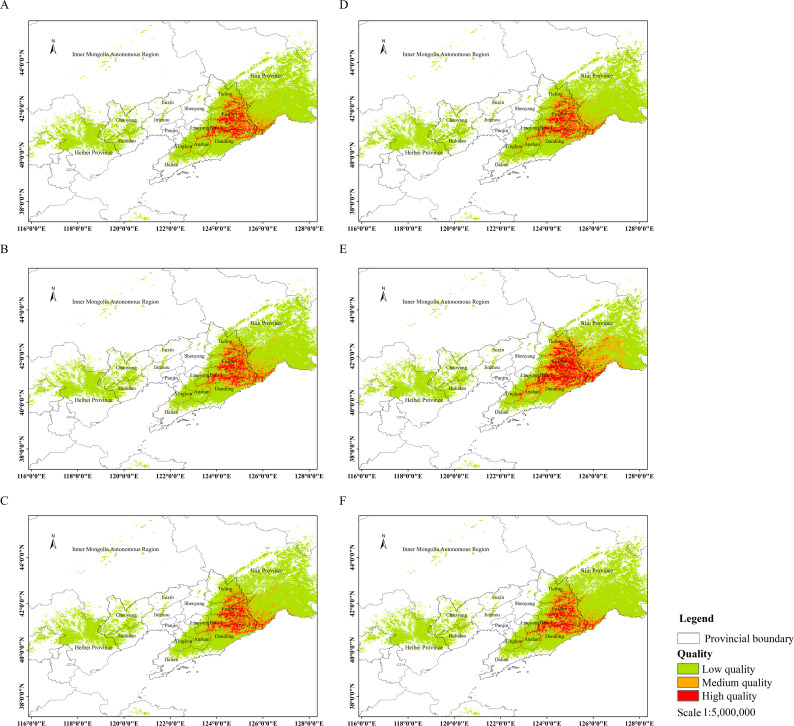
Predicted quality of *E. senticosus* in Liaoning Province under future climate conditions. Six future climate scenarios predicted based on the MAXENT-GD coupled model. **(A)** Quality under SSP1-2.6 in the 2050s. **(B)** Quality under SSP2-4.5 in the 2050s. **(C)** Quality under SSP5-8.5 in the 2050s. **(D)** Quality under SSP1-2.6 in the 2090s. **(E)** Quality under SSP2-4.5 in the 2090s. **(F)** Quality under SSP5-8.5 in the 2090s.

Based on the MAXENT and MAXENT-GD models, we constructed the comprehensive quality zoning of *E. senticosus* according to the above methods, and divided the study area into high-quality area (0.6–1.0), medium-quality area (0.4–0.6), and low-quality area (0.0–0.4) based on the comprehensive quality suitability value. On this basis, within the fixed suitable area range, this study evaluated the spatial dynamic changes of its internal comprehensive quality. The results showed that, compared with the MAXENT model, the MAXENT-GD model predicted a smaller area of high-quality and medium-quality areas, while the low-quality area was larger (**Figure 7**). Regarding the prediction of future comprehensive quality of *E. senticosus*, the results of the two models were generally consistent. Under different future climate scenarios, the total area of *E. senticosus* quality remained stable (10.7055 × 10^4^ km²), without significant change, but the internal composition of high-, medium-, and low-quality grades changed significantly (see **Table 4**, **Figures 8**, **9**). This indicates that future climate change primarily drives the spatial differentiation of quality within the suitable area, rather than the contraction or expansion of the suitable area range. Compared with the current climate scenario, the high-quality area showed a decreasing trend under future scenarios, while the low-quality area showed an increasing trend, indicating that climate change may have a negative impact on the habitat quality of *E. senticosus*, leading to the degradation of the original high-quality suitable areas. This means that the potential production areas of high-quality *E. senticosus* medicinal material will tend to shrink in the future, and the sustainable utilization of resources faces certain challenges. Therefore, while giving priority to protecting the existing high-quality areas, habitat restoration and adaptive management of medium- and low-quality areas should be strengthened to mitigate the trend of quality degradation and ensure the long-term sustainable utilization of *E. senticosus* resources.

**Table 4:** Results of quality area of *E. senticosus.*.

Model	Scenario	Area(×10^4^km^2^)		
MAXENT		L	M	H
	Now	4.51	2.70	3.49
	ssp126-2050s	4.87	2.77	3.07
	ssp245-2050s	5.03	2.72	2.96
	ssp585-2050s	5.07	2.71	2.93
	ssp126-2090s	4.83	2.77	3.10
	ssp245-2090s	4.90	2.82	2.98
	ssp585-2090s	4.81	2.75	3.15
MAXENT-GD		L	M	H
	Now	6.45	2.22	2.04
	ssp126-2050s	6.43	2.17	2.10
	ssp245-2050s	6.58	2.16	1.96
	ssp585-2050s	6.37	2.23	2.10
	ssp126-2090s	6.43	2.23	2.05
	ssp245-2090s	6.38	2.24	2.09
	ssp585-2090s	6.45	2.22	2.04

L: Low, M: Medium, H: High.

In summary, the ecological suitability areas and quality suitability areas of *E. senticosus* have a high degree of spatial consistency, which is consistent with existing theoretical understanding. The areas with higher comprehensive quality are mainly concentrated in Fushun City, Benxi City, and northern Dandong City. These areas are both the core areas of ecological suitability and the key development areas for future simulated wild cultivation of *E. senticosus* and the production of high-quality medicinal material.

## 4 Discussion

### 4.1 Main environmental variables of *E. senticosus*

From the results, it can be seen that precipitation, temperature, and geographical environment have significant effects on the accumulation of active components in *E. senticosus*. Adequate precipitation contributes to the accumulation of active components, and appropriately narrowing the seasonal temperature difference contributes to the accumulation of glycosides and phenolic acids in *E. senticosus*. This may be related to the fact that *E. senticosus* is a plant that prefers warm and humid climates and is cold-tolerant. Among all Environmental variables, precipitation accounted for 51% of the total weight. Precipitation modulates soil water availability and plant water status, activating the activities of key enzymes (PAL, CHS) in the phenylpropanoid metabolic pathway, and promoting the biosynthesis of phenolic acids, flavonoids, and coumarins secondary metabolites (Kuanchao et al., 2008; Biała and Jasiński, 2018; Jun et al., 2022). Eastern Liaoning Province, influenced by the oceanic monsoon climate, receives annual precipitation of more than 1000 mm, which is very suitable for the growth of *E. senticosus*. Vegetation type (zblx) accounted for 15.9% of all Environmental variables, indicating that the suitable habitat selection of *E. senticosus* has clear community specificity (Jinhui et al., 2022), with a particular preference for the temperate mixed coniferous and broad-leaved forest environment. This factor constitutes the microhabitat foundation for *E. senticosus* establishment by regulating understory light, soil organic matter, and interspecific competitive relationships. Compared with bare land, grassland, or pure coniferous forest, *E. senticosus* is more inclined to appear under mixed forests or at forest edges. Meanwhile, studies have shown that geographical environments such as soil, slope, and elevation also have a certain influence on the growth of *E. senticosus* (Yuan et al., 2025), with a comprehensive contribution rate of 9.0%. Humic cambisols are rich in organic matter, have good aggregate structure and water-air coordination capacity, which can not only provide a loose and aerated physical environment for the root development of *E. senticosus*, but also continuously supply nutrients through the adsorption and mineralization of humus. This soil type is widely distributed under temperate mixed coniferous and broad-leaved forests, highly consistent with the community habitat of *E. senticosus*, and together they constitute the geographical basis for its suitability distribution. Gentle slopes facilitate drainage and avoid waterlogging and root rot (Fang et al., 2021), which precisely matches the characteristic of *E. senticosus* of “preferring humidity but fearing waterlogging”. The combination of aspect and slope affects solar radiation reception and thus affects the understory microclimate environment, thereby being more suitable for the growth of *E. senticosus*. Studies have also shown that mid-altitude areas are the zones where *E. senticosus* community structure is the most stable and the coexistence relationship with other species is the most harmonious (Vásquez-Aguilar et al., 2025). *E. senticosus* is mostly distributed in mixed woodlands with higher altitude and less light. At mid-altitudes, the species and quantity of arbor layer trees reach their peak, forming a suitable shaded environment, allowing *E. senticosus* to obtain scattered light while avoiding fierce competition from sun-loving weeds. This is consistent with the slightly shade-tolerant characteristic of *E. senticosus*.

### 4.2 Changes in the suitable distribution of *E. senticosus*

This study is based on the joint prediction of the MAXENT model and the MAXENT-GD model. The results of the two models are highly consistent, both indicating that Fushun City, Benxi City, and northern Dandong City are the core distribution areas of high-quality zones of *E. senticosus*. This area is located in the traditional suitable growing area of *E. senticosus*, has a good resource base and ecological foundation, and can be used as a priority area for the cultivation and planting of *E. senticosus* in the current and next half century. The high-quality zones of *E. senticosus* are concentrated in the eastern Liaoning region. This spatial pattern is closely related to the unique topography and climatic conditions of this region. Eastern Liaoning is located in the transitional zone from the remaining mountain range of Changbai Mountain to the North China flora. The terrain is mainly composed of medium and low mountains, forming a complex mountain habitat structure. At the same time, this region is significantly regulated by the marine climate, characterized by warm and humid conditions and abundant precipitation, which well matches the ecological habits of *E. senticosus* that prefers a warm and humid climate and has a preference for certain altitudes.

The prediction results of the two models for *E. senticosus* show that under future climate scenarios, the total area of suitable zones for *E. senticosus* will decrease compared with the present, and the suitable zones show an overall degradation trend (Geng et al., 2022). Among them, the area in the 2050s decreases with increasing emission intensity, while in the 2090s it increases with increasing emission intensity. This trend is consistent with the research results on *Psittacanthus* species (Vásquez-Aguilar et al., 2025), and may be due to the migration of species distribution areas to high altitudes and high latitudes under the influence of climate in the 2090s (Solow et al., 2014; Li et al., 2023). However, limited by the complexity of ecological conditions and the practical constraints of field sample collection, this study has not yet been able to obtain more sample data. Subsequent research intends to carry out more comprehensive field investigations and sample collection work in other suitable growing areas of *E. senticosus* to further deepen the understanding of the growth habits and environmental response mechanisms of *E. senticosus*, and to provide a more solid scientific basis for its resource protection and artificial cultivation.

### 4.3 Application and recommendations of the model

Given that the MAXENT-GD model is superior to the single MAXENT model in prediction accuracy, a technical path for the synergistic application of the two models can be constructed. First, the MAXENT model is used to carry out preliminary screening of the research subject to identify the macro pattern of its potential suitable areas. On this basis, the MAXENT-GD model is further used for precise identification, achieving stepwise refinement and accuracy improvement of the suitable areas through model coupling. The synergy of the two models can provide a more scientific and reliable decision-making basis for the cultivation zoning and resource conservation of *E. senticosus*, and provide operable technical support for species suitability assessment and precision planting guidance. 

### 4.4 Limitations of the study

This study has the following limitations: First, the spatial resolution of 1.0 km × 1.0 km may be difficult to fully capture local microhabitat heterogeneity. Future research may consider introducing higher-resolution (e.g., 30 m) remote sensing data to further improve assessment accuracy. Second, the model did not incorporate biological factors such as interspecific competition, pollination, and symbiosis, which may have a certain impact on the prediction results of suitable areas for *E. senticosus*, especially in areas where community succession is relatively active. In the future, if multi-species coexistence models or relevant community ecology data can be integrated, this deficiency may be compensated to some extent. Third, this study only focused on the average trends under multiple scenarios and did not quantify the inherent uncertainty of the CMIP6 models. Subsequent research should be based on a multi-model ensemble framework, using multiple Global Climate Models (GCMs) rather than relying on a single model, to more comprehensively characterize the uncertainty of predictions. In summary, the conclusions of this paper only represent preliminary understandings under specific climate scenarios and model frameworks. Actual conservation decisions still need to be combined with field validation for cautious adjustment and dynamic revision.

## 5 Conclusion

This study, grounded in an integrative approach combining theoretical modeling with empirical investigation, systematically simulated the ecological suitability and quality-based distribution patterns of *E. senticosus* under both current and future climate scenarios using the MAXENT and MAXENT-GD models. Notably, the MAXENT-GD model—by incorporating factor interaction analysis and risk detection mechanisms from geographical detectors—outperforms the standalone MAXENT model in both predictive accuracy and ecological interpretability, yielding projections that more closely align with actual habitat conditions. This framework provides an intuitive and scientifically robust basis for the conservation and spatial optimization of suitable habitats for *E. senticosus*. Our findings demonstrate that the distribution of *E. senticosus* is jointly governed by multiple environmental drivers, including precipitation, temperature, soil type, topography, and vegetation type, whereas the accumulation of its medicinal components exhibits specific responses to vegetation type, precipitation regime, and soil properties. Both models consistently project a degradation trend in suitable habitats for *E. senticosus* over the coming decades. In response, we advocate for the establishment of a dynamic planting suitability zoning mechanism, the designation of high-priority conservation zones centered on Fushun, Benxi, and northern Dandong, and the strategic deployment of simulated wild cultivation and elite seedling propagation bases within these areas. Furthermore, future climate projections should be explicitly integrated into industrial planning to ensure the sustainable utilization of *E. senticosus* resources and the high-quality development of the associated industry.

The principal innovation of this study lies in the integration of MAXENT modeling, UPLC, and geographical detector methods into a comprehensive analytical framework capable of predicting current and future ecological suitability and quality distribution for a target species. In addition, the use of multiple independent validation metrics substantially enhances the reliability of our model outputs. Collectively, this approach offers a transferable methodological template for analogous studies on other species and in other regions.

## Data Availability

The raw data supporting the conclusions of this article will be made available by the authors, without undue reservation.
