# Supplementary material for: Evaluation of ecological suitability and quality of wild Eleutherococcus senticosus in Liaoning, China
Source: Front Plant Sci. 2026 Jun 30;17:1849106. doi: 10.3389/fpls.2026.1849106 (PMC13364862; doi:10.3389/fpls.2026.1849106)

**Table S1.** Distribution points of *Eleutherococcus senticosus* in Liaoning and adjacent regions, and sampling information in Liaoning Province.

| No. | Area Name | Longitude | Latitude | Altitude | Stems |
| --- | --- | --- | --- | --- | --- |
| 1 | Liaoning Province, Tieling City, Changtu County, Dachegou | 124.4181°E | 42.9450°N | 285m | - |
| 2 | Liaoning Province, Tieling City, Changtu County, Shenshushan | 124.2517°E | 42.9031°N | 234m | - |
| 3 | Liaoning Province, Tieling City, Xifeng County, Xinyouling | 124.7601°E | 42.5277°N | 446m | 1 |
| 4 | Liaoning Province, Tieling City, Kaiyuan City, Sandao Bei | 124.6616°E | 42.4898°N | 282m | 2 |
| 5 | Liaoning Province, Fushun City, Qingyuan Manchu Autonomous County, Gunzigou | 125.0498°E | 42.3558°N | 369m | 0 |
| 6 | Liaoning Province, Fushun City, Qingyuan Manchu Autonomous County, Laohudingzi | 124.9486°E | 42.2441°N | 692m | 3 |
| 7 | Liaoning Province, Fushun City, Qingyuan Manchu Autonomous County, Baniugou | 124.8997°E | 42.1769°N | 478m | 3 |
| 8 | Liaoning Province, Fushun City, Qingyuan Manchu Autonomous County, Youfanggou | 124.8874°E | 41.9249°N | 479m | 1 |
| 9 | Liaoning Province, Fushun City, Qingyuan Manchu Autonomous County, Dayanggou | 125.1814°E | 41.9959°N | 535m | 7 |
| 10 | Liaoning Province, Fushun City, Shuncheng District, Huangdingshan Forest Park | 123.9080°E | 42.0183°N | 246m | - |
| 11 | Liaoning Province, Fushun City, Dongzhou District, Suihua Road East Section | 124.0882°E | 41.8931°N | 99m | 0 |
| 12 | Liaoning Province, Fushun City, San Kuaishi National Forest Park | 124.4160°E | 41.6698°N | 783m | 0 |
| 13 | Liaoning Province, Fushun City, Xinbin Manchu Autonomous County, Yangzigou | 125.2144°E | 41.7407°N | 494m | 3 |
| 14 | Liaoning Province, Fushun City, Xinbin Manchu Autonomous County, Madaogou | 125.3958°E | 41.6138°N | 579m | 4 |
| 15 | Liaoning Province, Fushun City, Xinbin Manchu Autonomous County, Haozigou | 124.9746°E | 41.5673°N | 591m | 3 |
| 16 | Liaoning Province, Fushun City, Xinbin Manchu Autonomous County, Dongcha | 124.6340°E | 41.4846°N | 472m | 0 |
| 17 | Liaoning Province, Fushun City, Xinbin Manchu Autonomous County, Shansongcun | 124.4486°E | 41.4485°N | 429m | 2 |
| 18 | Liaoning Province, Benxi City, Huanren Manchu Autonomous County, Donggou | 125.3026°E | 41.4648°N | 549m | 2 |
| 19 | Liaoning Province, Benxi City, Huanren Manchu Autonomous County, Qiangou | 124.9764°E | 41.3949°N | 446m | - |
| 20 | Liaoning Province, Benxi City, Huanren Manchu Autonomous County, Laotudingzi Mountain | 124.8902°E | 41.3177°N | 922m | - |
| 21 | Liaoning Province, Benxi City, Huanren Manchu Autonomous County, Jianjiaogou | 125.4503°E | 41.2234°N | 393m | 2 |
| 22 | Liaoning Province, Benxi City, Huanren Manchu Autonomous County, Heping Road | 125.5016°E | 41.1725°N | 452m | - |
| 23 | Liaoning Province, Benxi City, Huanren Manchu Autonomous County, Yaoqianshugou | 125.7688°E | 41.1639°N | 731m | - |
| 24 | Liaoning Province, Benxi City, Huanren Manchu Autonomous County, Yushugou | 125.6364°E | 40.9848°N | 579m | - |
| 25 | Liaoning Province, Benxi City, Nanfen District, Laomuling | 123.8160°E | 41.2431°N | 544m | 2 |
| 26 | Liaoning Province, Benxi City, Nanfen District, Shihugou | 123.9753°E | 41.1746°N | 796m | 0 |
| 27 | Liaoning Province, Benxi City, Nanfen District, Xiamatang Town, Sidaogou | 123.6852°E | 41.0392°N | 560m | 1 |
| 28 | Liaoning Province, Benxi City, Nanfen District, Gangcaodian | 123.8547°E | 41.0050°N | 455m | 2 |
| 29 | Liaoning Province, Benxi City, Nanfen District, Xiamatang Town, Xinjian Shiligou | 123.8906°E | 41.0183°N | 461m | 1 |
| 30 | Liaoning Province, Benxi City, Benxi Manchu Autonomous County, Yanghugou Forest Farm | 124.6698°E | 41.2012°N | 537m | 2 |
| 31 | Liaoning Province, Benxi City, Benxi Manchu Autonomous County, Daqinggoumen | 124.2271°E | 41.1111°N | 562m | - |
| 32 | Liaoning Province, Dandong City, Kuandian Manchu Autonomous County, Heigou | 125.1639°E | 41.0977°N | 514m | - |
| 33 | Liaoning Province, Dandong City, Kuandian Manchu Autonomous County, Tianhuashan Park | 124.5671°E | 41.0852°N | 628m | 2 |
| 34 | Liaoning Province, Dandong City, Kuandian Manchu Autonomous County, Xiaoyanggou | 124.7577°E | 41.0331°N | 462m | - |
| 35 | Kuandian Manchu Autonomous County, Shihu Township, Lazicun | 124.7600°E | 40.7800°N | 335m | 1 |
| 36 | Liaoning Province, Dandong City, Kuandian Manchu Autonomous County, Eryanggou | 124.8487°E | 40.7950°N | 408m | - |
| 37 | Liaoning Province, Dandong City, Fengcheng City, Dawanggou | 124.3742°E | 41.0500°N | 731m | - |
| 38 | Liaoning Province, Dandong City, Fengcheng City, Diaowolazi | 124.2670°E | 41.0291°N | 380m | 1 |
| 39 | Liaoning Province, Dandong City, Fengcheng City, Lingqianpuzi | 124.1595°E | 40.1724°N | 218m | - |
| 40 | Liaoning Province, Liaoyang City, Dengta City, Yangjiagou | 123.6415°E | 41.3146°N | 158m | - |
| 41 | Liaoning Province, Benxi City, Pingshan District, Ligou Erzu | 123.6489°E | 41.1576°N | 317m | 1 |
| 42 | Liaoning Province, Liaoyang City, Gongchangling District, Laolinziding | 123.4381°E | 41.1000°N | 470m | - |
| 43 | Liaoning Province, Anshan City, Tiedong District, Qianshan | 123.1276°E | 40.9929°N | 647m | 1 |
| 44 | Liaoning Province, Anshan City, Xiuyan Manchu Autonomous County, Shiren Donggou | 123.7316°E | 40.5304°N | 435m | - |
| 45 | Liaoning Province, Dalian City, Zhuanghe City, Xianrendong Town, Xianrendong Forest Park | 122.9471°E | 40.0188°N | 177m | - |
| 46 | Jilin Province, Baishan City, Fusong County | 127.3123°E | 42.3313°N | 577m | - |
| 47 | Jilin Province, Baishan City, Jiangyuan District, Daheisonggou | 126.6707°E | 41.9061°N | 514m | - |
| 48 | Jilin Province, Baishan City, Linjiang City, Jilin Ganfanpen Forest Park | 126.9465°E | 41.8460°N | 631m | - |
| 49 | Inner Mongolia Autonomous Region, Chifeng City, Kalaqin Town, Maojingba National Forest Park | 118.2496°E | 41.6174°N | 419m | - |
| 50 | Jilin Province, Tonghua City, Dongchang District, Baijiyao National Forest Park | 126.0901°E | 41.5661°N | 822m | - |
| 51 | Jilin Province, Baishan City, Changbai Korean Autonomous County | 128.0581°E | 41.5502°N | 1128m | - |
| 52 | Jilin Province, Baishan City, Changbai Korean Autonomous County | 127.9422°E | 41.4746°N | 884m | - |
| 53 | Hebei Province, Chengde City, Xinglong County, Wuling Mountain | 117.4649°E | 40.6063°N | 1291m | - |
| 54 | Hebei Province, Chengde City, Xinglong County | 117.7455°E | 40.4570°N | 671m | - |
| Total |  |  |  |  | 47 |

**Table S2.** Table of sample IDs used in this study

| Region | Sample ID |
| --- | --- |
| Xifeng County, Tieling City, Liaoning Province | 21122320250825001 |
| Kaiyuan City, Tieling City, Liaoning Province | 21128220250825001 |
| Qingyuan County, Fushun City, Liaoning Province | 21042320250823001 |
| Fushun County, Fushun City, Liaoning Province | 210421140820102LY |
| Shuncheng District, Fushun City, Liaoning Province | 210411190923070LY |
| Xinbin County, Fushun City, Liaoning Province | 210422150709011LY |
| Huanren County, Benxi City, Liaoning Province | 21052220250818001 |
| Nanfen District, Benxi City, Liaoning Province | 210505190726944LY |
| Benxi County, Benxi City, Liaoning Province | 210521150819224LY |
| Pingshan District, Benxi City, Liaoning Province | 21050220250813001 |
| Kuandian County, Dandong City, Liaoning Province | 21062420250701001 |
| Fengcheng City, Dandong City, Liaoning Province | 21060620250826001 |
| Tiedong District, Anshan City, Liaoning Provinc | 21030220250901001 |

Note: The sample IDs in some regions are shared with those from the National Survey of

Chinese Medicine Resources, with the suffix "LY" added to the IDs.

**Table S3.** Current Environmental Variables.

| Environmental factor | Abbreviation | Variable description | Unit |
| --- | --- | --- | --- |
| Climatic factors | prec1~prec12 | Monthly average precipitation (Jan-Dec) | mm |
|  | tmean1~tmean12 | Monthly average temperature (Jan-Dec) | ℃ * 10 |
|  | bio1 | Annual average temperature | ℃ * 10 |
|  | bio2 | Mean diurnal range | ℃ * 10 |
|  | bio3 | Isothermality | - |
|  | bio4 | Temperature seasonality (standard deviation) | ℃ * 10 |
|  | bio5 | Max temperature of warmest month | ℃ * 10 |
|  | bio6 | Min temperature of coldest month | ℃ * 10 |
|  | bio7 | Temperature annual range | ℃ * 10 |
|  | bio8 | Average temperature of wettest quarter | ℃ * 10 |
|  | bio9 | Average temperature of driest quarter | ℃ * 10 |
|  | bio10 | Average temperature of warmest quarter | ℃ * 10 |
|  | bio11 | Average temperature of coldest quarter | ℃ * 10 |
|  | bio12 | Annual precipitation | mm |
|  | bio13 | Precipitation of wettest month | mm |
|  | bio14 | Precipitation of driest month | mm |
|  | bio15 | Precipitation seasonality | - |
|  | bio16 | Precipitation of wettest quarter | mm |
|  | bio17 | Precipitation of driest quarter | mm |
|  | bio18 | Precipitation of warmest quarter | mm |
|  | bio19 | Precipitation of coldest quarter | mm |
| Soil factors | ph | PH | - |
|  | trylzjhnl | Soil cation exchange capacity | cmol/kg |
|  | trhsl | Soil sand content | % |
|  | trntl | Soil clay content | % |
|  | SoilType | Soil subgroup（sym90） | - |
|  | tryxshldj | Soil available water content class | - |
|  | trzdfl | Soil texture class | - |
|  | tryjthl | Soil organic carbon content | % |
| Topographic factors | altitude | Elevation | m |
|  | slope | Slope | ° |
|  | aspect | Aspect | - |
| Vegetation type | zblx | Vegetation type | - |
| Kira index | index_ci | Warmth index | - |
|  | index_hi | Coldness index | - |
|  | index_wi | Aridity index | - |
| Integrated climatic factors | wc2.1_30s_srad_01~12 | Monthly radiation intensity | - |
|  | wc2.1_30s_tmax_01~12 | Monthly maximum temperature | - |
|  | wc2.1_30s_tmin_01~12 | Monthly minimum temperature | - |
|  | wc2.1_30s_vapr_01~12 | Monthly water vapor pressure | - |

**Table S4.** Future Environmental Variables.

| Period | Scenario | Factor | Description |
| --- | --- | --- | --- |
| 2050s | SSP126 | prec1~prec12（mm） | Monthly mean precipitation (Jan-Dec) |
|  |  | wc2.1_30s_tmax_01~12 - | Monthly maximum temperature |
|  |  | wc2.1_30s_tmin_01~12 - | Monthly minimum temperature |
|  | SSP245 | bio1（℃ * 10） | Annual mean temperature |
|  |  | bio2（℃ * 10） | Mean diurnal range |
|  |  | bio3 - | Isothermality |
|  |  | bio4 - | Temperature seasonality (standard deviation) |
|  | SSP585 | bio5（℃ * 10） | Max temperature of warmest month |
|  |  | bio6（℃ * 10） | Min temperature of coldest month |
|  |  | bio7（℃ * 10） | Temperature annual range |
|  |  | bio8（℃ * 10） | Mean temperature of wettest quarter |
| 2090s | SSP126 | bio9（℃ * 10） | Mean temperature of driest quarter |
|  |  | bio10（℃ * 10） | Mean temperature of warmest quarter |
|  |  | bio11（℃ * 10） | Mean temperature of coldest quarter |
|  |  | bio12（mm） | Annual precipitation |
|  | SSP245 | bio13（mm） | Precipitation of wettest month |
|  |  | bio14（mm） | Precipitation of driest month |
|  |  | bio15 - | Precipitation seasonality (coefficient of variation) |
|  | SSP585 | bio16（mm） | Precipitation of wettest quarter |
|  |  | bio17（mm） | Precipitation of driest quarter |
|  |  | bio18（mm） | Precipitation of warmest quarter |
|  |  | bio19（mm） | Precipitation of coldest quarter |

**Table S5.** Results of autocorrelation among environmental variables

| Variable | prec7 | prec11 | zblx | index_ci | bio4 | soiltype | tmean11 | aspect | altitude | slope |
| --- | --- | --- | --- | --- | --- | --- | --- | --- | --- | --- |
| prec7 | 1 | 0.796** | 0.167 | 0.16 | -0.423** | 0.155 | 0.256 | -0.019 | 0.138 | 0.217 |
| prec11 | 0.796** | 1 | 0.175 | 0.082 | -0.175 | 0.117 | 0.161 | -0.081 | 0.003 | -0.056 |
| zblx | 0.167 | 0.175 | 1 | -0.098 | 0.01 | 0.032 | -0.079 | -0.161 | 0.102 | 0.05 |
| index_ci | 0.16 | 0.082 | -0.098 | 1 | -0.630** | 0.118 | 0.788** | 0.142 | -0.734** | -0.175 |
| bio4 | -0.423** | -0.175 | 0.01 | -0.630** | 1 | 0.09 | -0.524** | -0.051 | 0.023 | -0.196 |
| soiltype | 0.155 | 0.117 | 0.032 | 0.118 | 0.09 | 1 | 0.243 | 0.258 | -0.259 | -0.157 |
| tmean11 | 0.256 | 0.161 | -0.079 | 0.788** | -0.524** | 0.243 | 1 | 0.256 | -0.612** | -0.221 |
| aspect | -0.019 | -0.081 | -0.161 | 0.142 | -0.051 | 0.258 | 0.256 | 1 | -0.175 | 0.043 |
| altitude | 0.138 | 0.003 | 0.102 | -0.734** | 0.023 | -0.259 | -0.612** | -0.175 | 1 | 0.394** |
| slope | 0.217 | -0.056 | 0.05 | -0.175 | -0.196 | -0.157 | -0.221 | 0.043 | 0.394** | 1 |

Note: *P<0.05,**P<0.01

**Table S6.** Extracted values of environmental variables

| No. | prec7 | prec11 | zblx | index_ci | bio4 | soiltype | tmean11 | aspect | altitude | slope |
| --- | --- | --- | --- | --- | --- | --- | --- | --- | --- | --- |
| 1 | 259 | 29 | 32 | -61.3 | 11641 | 28 | -15 | 8 | 630 | 7.42327 |
| 2 | 198 | 23 | 29 | -53.5 | 12639 | 45 | 0 | 8 | 92 | 0.89926 |
| 3 | 193 | 25 | 32 | -61 | 12865 | 72 | -15 | 9 | 365 | 2.01794 |
| 4 | 244 | 28 | 36 | -81.7 | 12814 | 72 | -50 | 2 | 1026 | 7.32403 |
| 5 | 231 | 32 | 19 | -70.3 | 12878 | 72 | -29 | 7 | 538 | 2.11378 |
| 6 | 241 | 30 | 32 | -65.8 | 12693 | 82 | -20 | 9 | 462 | 1.69830 |
| 7 | 252 | 30 | 32 | -71.4 | 12620 | 72 | -32 | 7 | 717 | 2.25460 |
| 8 | 236 | 31 | 32 | -69.3 | 12800 | 72 | -27 | 7 | 517 | 2.70547 |
| 9 | 219 | 28 | 19 | -73.6 | 13083 | 72 | -35 | 3 | 585 | 1.19559 |
| 10 | 216 | 29 | 32 | -67.5 | 12902 | 45 | -24 | 2 | 452 | 1.11477 |
| 11 | 198 | 24 | 32 | -68.9 | 13179 | 45 | -26 | 5 | 370 | 1.02793 |
| 12 | 206 | 23 | 32 | -72.1 | 13143 | 72 | -32 | 4 | 524 | 4.44167 |
| 13 | 229 | 23 | 32 | -58.4 | 12116 | 72 | -27 | 9 | 407 | 4.23431 |
| 14 | 241 | 23 | 20 | -69.3 | 12344 | 72 | -11 | 9 | 784 | 6.89263 |
| 15 | 251 | 28 | 32 | -67.7 | 12250 | 72 | -31 | 9 | 710 | 2.55300 |
| 16 | 265 | 28 | 32 | -65.1 | 12187 | 72 | -32 | 5 | 597 | 4.53696 |
| 17 | 271 | 28 | 32 | -64.2 | 12319 | 72 | -18 | 7 | 527 | 5.34091 |
| 18 | 261 | 33 | 19 | -64.1 | 12435 | 72 | -17 | 9 | 474 | 5.05842 |
| 19 | 264 | 33 | 20 | -64.7 | 12449 | 72 | -27 | 9 | 508 | 3.41739 |
| 20 | 250 | 33 | 32 | -69.3 | 12670 | 72 | -27 | 2 | 579 | 3.54673 |
| 21 | 276 | 32 | 19 | -89.2 | 12576 | 12 | -59 | 4 | 1240 | 5.86344 |
| 22 | 258 | 33 | 32 | -68.1 | 12543 | 72 | -25 | 3 | 591 | 3.00911 |
| 23 | 266 | 34 | 32 | -73.2 | 12561 | 72 | -25 | 9 | 777 | 7.32142 |
| 24 | 267 | 33 | 32 | -55.7 | 12097 | 72 | -3 | 7 | 296 | 0.90995 |
| 25 | 305 | 33 | 32 | -62.6 | 12011 | 72 | -14 | 4 | 579 | 2.89283 |
| 26 | 286 | 36 | 19 | -60.2 | 12198 | 72 | -15 | 8 | 423 | 4.33473 |
| 27 | 280 | 32 | 36 | -70.8 | 12283 | 72 | -9 | 6 | 795 | 0.71178 |
| 28 | 283 | 31 | 36 | -72.9 | 12404 | 72 | -35 | 6 | 839 | 6.96792 |
| 29 | 279 | 30 | 32 | -47.8 | 11310 | 72 | 11 | 6 | 243 | 3.97252 |
| 30 | 269 | 33 | 32 | -57.7 | 12037 | 82 | -7 | 4 | 329 | 0.53120 |
| 31 | 276 | 31 | 36 | -67.1 | 12214 | 72 | -24 | 6 | 672 | 6.55170 |
| 32 | 221 | 31 | 32 | -58.4 | 12103 | 72 | -25 | 3 | 447 | 4.17266 |
| 33 | 214 | 31 | 32 | -52.4 | 12177 | 72 | 1 | 8 | 180 | 3.32319 |
| 34 | 192 | 21 | 32 | -66.5 | 13185 | 82 | -23 | 9 | 291 | 2.42968 |
| 35 | 183 | 16 | 20 | -64 | 13114 | 82 | -24 | 9 | 218 | 2.50405 |
| 36 | 187 | 15 | 32 | -67.7 | 13207 | 72 | -23 | 8 | 309 | 3.18717 |
| 37 | 193 | 21 | 32 | -67 | 13142 | 72 | -24 | 7 | 338 | 2.57939 |
| 38 | 211 | 19 | 29 | -57.8 | 12069 | 28 | -28 | 2 | 462 | 5.30130 |
| 39 | 216 | 18 | 13 | -51.7 | 11304 | 45 | 4 | 9 | 484 | 2.30052 |
| 40 | 239 | 20 | 32 | -79 | 13024 | 72 | -45 | 7 | 777 | 8.48378 |
| 41 | 212 | 19 | 19 | -82.1 | 13405 | 72 | -36 | 5 | 712 | 5.29394 |
| 42 | 205 | 7 | 27 | -44.5 | 11348 | 73 | 6 | 2 | 516 | 2.50168 |
| 43 | 149 | 7 | 32 | -66.3 | 11638 | 28 | -36 | 5 | 1115 | 3.06878 |
| 44 | 194 | 7 | 19 | -74.3 | 11249 | 72 | -24 | 9 | 1509 | 13.1676 |
| 45 | 169 | 23 | 32 | -88.9 | 12492 | 53 | -59 | 4 | 1144 | 1.28372 |
| 46 | 226 | 27 | 32 | -91.3 | 13324 | 72 | -50 | 6 | 967 | 4.98528 |
| 47 | 168 | 24 | 33 | -73.6 | 12739 | 24 | -50 | 6 | 634 | 4.40873 |
| 48 | 192 | 23 | 32 | -85.7 | 13401 | 72 | -36 | 9 | 741 | 2.17138 |
| 49 | 251 | 27 | 32 | -59.8 | 11991 | 82 | -13 | 9 | 411 | 2.74668 |
| 50 | 239 | 26 | 32 | -60.8 | 12051 | 28 | -13 | 5 | 489 | 6.1996 |
| 51 | 307 | 36 | 32 | -59.1 | 11955 | 72 | -7 | 8 | 463 | 2.18826 |
| 52 | 249 | 27 | 32 | -58.7 | 11937 | 72 | -11 | 8 | 386 | 8.72696 |
| 53 | 245 | 29 | 32 | -63.4 | 12466 | 72 | -10 | 3 | 457 | 3.08917 |
| 54 | 204 | 25 | 20 | -69.4 | 13085 | 72 | -27 | 3 | 454 | 1.83959 |

**Table S7.** Results of content determination

| No. | Syringin | 3,4-Dihydroxybenzoic acid | Eleutheroside E | Hyperoside | Isofraxidin | Chlorogenic acid |
| --- | --- | --- | --- | --- | --- | --- |
| C01 | 83.22±5.84 | 119.72±0.37 | 889.66±8.37 | 43.72±0.74 | 142.38±0.83 | 5199.84±187.34 |
| C02 | 142.99±2.47 | 34.36±2.14 | 852.23±2.89 | 26.22±1.58 | 490.43±0.27 | 1033.87±52.17 |
| C03 | 202.07±19.36 | 34.32±0.82 | 580.43±5.27 | 35.77±1.33 | 461.46±0.94 | 967.60±289.46 |
| C04 | 140.72±2.89 | 86.85±1.56 | 597.84±21.54 | 40.60±0.29 | 241.86±0.32 | 1458.47±13.85 |
| C05 | 231.97±12.57 | 76.69±0.43 | 705.95±2.14 | 48.09±1.91 | 289.24±0.67 | 1436.75±94.23 |
| C06 | 122.25±2.32 | 81.22±0.69 | 582.15±4.93 | 42.95±1.57 | 136.59±0.18 | 1022.85±276.58 |
| C07 | 113.08±27.48 | 35.89±0.28 | 693.74±14.68 | 29.56±0.46 | 72.44±0.49 | 359.61±0.67 |
| C08 | 99.40±2.71 | 72.92±2.87 | 526.19±3.21 | 20.35±2.13 | 89.70±0.76 | 1524.52±168.92 |
| C09 | 194.58±8.63 | 78.73±0.91 | 671.77±2.56 | 27.86±1.22 | 100.66±0.23 | 1640.90±234.71 |
| C10 | 184.04±2.54 | 111.69±0.54 | 828.67±5.79 | 54.60±0.85 | 260.03±0.39 | 1662.97±41.39 |
| C11 | 105.94±21.95 | 101.22±0.73 | 593.74±18.42 | 50.68±2.69 | 166.94±0.58 | 1690.44±207.53 |
| C12 | 307.73±2.17 | 58.52±1.23 | 1007.07±2.47 | 37.83±0.17 | 104.26±0.12 | 912.88±118.64 |
| C13 | 345.83±29.36 | 73.91±0.39 | 1063.54±26.31 | 45.21±1.48 | 114.18±0.85 | 1026.27±295.27 |
| C14 | 347.38±2.82 | 96.30±0.66 | 943.69±3.85 | 110.04±2.34 | 307.14±0.44 | 1891.78±11.29 |
| C15 | 418.22±4.79 | 107.26±2.45 | 1335.44±2.19 | 111.49±3.63 | 345.41±0.29 | 1858.08±76.48 |
| C16 | 154.19±2.63 | 31.04±0.18 | 508.96±5.32 | 23.42±0.51 | 87.12±0.91 | 362.21±195.13 |
| C17 | 81.00±15.42 | 127.84±0.52 | 815.12±11.76 | 62.09±2.87 | 176.55±0.36 | 1129.81±263.89 |
| C18 | 55.80±2.96 | 93.71±0.87 | 643.53±2.63 | 40.91±0.38 | 146.98±0.63 | 1119.40±22.67 |
| C19 | 149.95±11.28 | 46.16±0.31 | 881.83±4.18 | 36.42±1.29 | 117.11±0.16 | 1331.76±143.56 |
| C20 | 341.22±2.39 | 95.43±1.78 | 1103.85±23.59 | 74.56±2.76 | 159.45±0.47 | 1219.95±281.74 |
| C21 | 17.44±24.57 | 44.29±0.94 | 934.84±3.47 | 45.50±3.92 | 154.17±0.72 | 623.66±210.93 |
| C22 | 52.38±2.15 | 75.68±0.47 | 309.96±2.91 | 26.01±0.44 | 149.06±0.21 | 646.20±179.25 |
| C23 | 144.85±7.68 | 54.21±2.68 | 739.22±5.64 | 53.55±2.19 | 146.27±0.38 | 1196.03±248.36 |
| C24 | 72.10±2.74 | 76.74±0.23 | 369.25±16.83 | 32.38±0.54 | 164.73±0.95 | 628.72±61.58 |
| C25 | 269.77±18.93 | 88.46±0.76 | 886.61±2.38 | 24.83±1.39 | 390.09±0.42 | 875.07±132.79 |
| C26 | - | 28.59±0.15 | 39.08±4.72 | 4.86±0.62 | 27.10±0.27 | 132.21±298.61 |
| C27 | 89.96±3.57 | 39.89±0.62 | 506.39±9.25 | 24.41±0.23 | 117.80±0.79 | 714.62±0.83 |
| C28 | 121.72±2.81 | 72.01±0.89 | 693.95±2.15 | 34.45±1.56 | 156.11±0.14 | 849.19±156.47 |
| C29 | 364.07±22.64 | 43.22±1.92 | 1043.98±5.98 | 52.03±2.48 | 240.45±0.53 | 1404.92±214.38 |
| C30 | 221.93±2.46 | 57.54±0.34 | 1365.09±19.67 | 55.01±0.71 | 218.75±0.88 | 1288.22±35.29 |
| C31 | 204.98±9.53 | 40.68±0.58 | 851.14±3.54 | 30.96±1.27 | 170.87±0.33 | 1010.26±107.64 |
| C32 | 188.32±2.68 | 43.91±0.71 | 868.60±2.77 | 28.93±2.95 | 243.22±0.46 | 983.41±269.83 |
| C33 | 49.39±14.39 | 39.51±0.26 | 358.40±6.43 | 17.39±2.49 | 70.62±0.68 | 365.86±21.17 |
| C34 | 258.53±2.19 | 48.34±2.31 | 1089.72±24.82 | 27.79±1.35 | 233.64±0.19 | 252.88±88.56 |
| C35 | 110.96±26.82 | 31.80±0.49 | 388.50±2.29 | - | 156.55±0.52 | 653.35±203.47 |
| C36 | 137.50±2.92 | 56.18±0.83 | 852.98±5.11 | 43.88±0.16 | 190.57±0.81 | 1354.74±127.39 |
| C37 | 95.88±6.47 | 28.12±0.21 | 485.74±13.46 | 24.61±1.42 | 131.79±0.37 | 655.20±257.62 |
| C38 | 41.66±2.51 | 61.92±0.67 | 512.55±3.69 | 26.98±1.57 | 108.38±0.64 | 941.80±15.73 |
| C39 | 159.67±20.73 | 97.12±0.44 | 721.11±2.84 | 36.19±0.81 | 134.44±0.11 | 975.33±71.29 |
| C40 | 44.90±2.38 | 42.72±1.09 | 382.95±4.57 | 14.24±1.18 | 128.41±0.59 | 251.13±192.84 |
| C41 | 247.85±28.61 | 88.66±0.92 | 766.87±22.91 | 54.78±2.39 | 144.71±0.73 | 1847.54±284.37 |
| C42 | 338.27±2.83 | 90.90±0.57 | 831.01±2.96 | 63.56±0.67 | 151.10±0.24 | 1751.30±45.98 |
| C43 | 212.15±13.56 | 78.00±1.45 | 609.83±5.48 | 62.68±1.53 | 162.15±0.48 | 1258.18±164.73 |
| C44 | 114.43±2.97 | 61.10±0.79 | 650.24±17.35 | 25.23±0.74 | 80.92±0.86 | 720.76±310.56 |
| C45 | 119.85±17.84 | 92.17±0.33 | 616.86±3.12 | 47.30±0.28 | 165.08±0.31 | 1086.36±0.42 |
| C46 | 225.51±2.62 | 114.74±0.85 | 1001.62±2.63 | 64.23±1.47 | 168.29±0.99 | 1979.95±98.67 |
| C47 | 186.77±30.00 | 68.86±0.29 | 983.71±27.00 | 72.14±2.96 | 205.09±0.56 | 671.99±211.59 |
| Average | 168.35 | 68.70 | 737.99 | 41.62 | 179.15 | 1148.27 |

**Table S8.** Results of linear relationship investigation

| Chemical composition | Regression equation | r | Linear range/μg·mL^-1^ | LOQ/μg·mL^-1^ | LOD/μg·mL^-1^ |
| --- | --- | --- | --- | --- | --- |
| syringin | y=5854.9x-3.3414 | 0.9996 | 2.181~109.091 | 0.00627 | 0.00188 |
| 3,4-Dihydroxybenzoic acid | y=8038.6x-19.823 | 0.9999 | 4.242~212.121 | 0.0156 | 0.00469 |
| eleutheroside E | y=140.71x-0.9166 | 0.9996 | 29.091~232.727 | 0.232 | 0.0695 |
| hyperoside | y=4063.6x-3.2914 | 0.9998 | 2.181~54.545 | 0.00847 | 0.00254 |
| isofraxidin | y=1133.4x-5.1186 | 0.9999 | 8.727~436.363 | 0.0378 | 0.0113 |
| chlorogenic acid | y=7231.6x-183.79 | 0.9996 | 7.272~1236.363 | 0.00309 | 0.000927 |

**Table S9.** Correlation analysis between effective components and environmental factors.

| Environmental variable | Syringin | 3,4-Dihydroxybenzoic acid | Eleutheroside E | Hyperoside | Isofraxidin | Chlorogenic acid |
| --- | --- | --- | --- | --- | --- | --- |
| prec7 |  | 0.308* |  | 0.298* |  | 0.317* |
| prec11 |  | 0.302* |  | 0.292* |  |  |
| zbl*x* | 0.306* | 0.318* |  |  |  |  |
| bio4 |  |  |  | -0.288* |  | -0.313* |
| soilt*y*pe |  |  | 0.345* |  |  |  |
| slope |  |  |  |  | 0.479** |  |

Note: **P*<0.05,***P*<0.01

**Table S10.** Results of the regression equation for quality zoning

| Component | Regression equation | p-value | R² | Q² |
| --- | --- | --- | --- | --- |
| Syringin | Y = 0.0034034 + 0.0004885 zblx | 5.23×10⁻¹¹< 0.005 | 0.6138 | 0.5892 |
| 3,4-Dihydroxybenzoic acid | Y = -0.0236 + 0.0000331 prec7 + 0.000151 prec11 + 0.000412 zblx | 1.23×10⁻¹⁸< 0.005 | 0.8652 | 0.817 |
| Eleutheroside E | Y = 0.0309335 + 0.0006052 soiltype | 7.65×10⁻⁷< 0.005 | 0.4158 | 0.3876 |
| Hyperoside | Y = 0.0041623 - 0.0018429 prec7 + 0.0016899 prec11 - 0.0012265 bio4 | 2.34×10⁻¹⁴< 0.005 | 0.7548 | 0.7123 |
| Isofraxidin | Y = 0.0111829 + 0.0021380 slope | 6.54×10⁻⁶< 0.005 | 0.3642 | 0.3318 |
| Chlorogenic acid | Y = 0.1148273 - 0.0099075 prec7 - 0.0294874 bio4 | 5.87×10⁻⁷< 0.005 | 0.4783 | 0.4295 |

**Table S11.** Judgment matrix scoring table

| Component | syringin | 3,4-Dihydroxybenzoic acid | eleutheroside E | hyperoside | isofraxidin | chlorogenic acid |
| --- | --- | --- | --- | --- | --- | --- |
| syringin | 1 | 3.333 | 1.250 | 1.250 | 1.667 | 1.429 |
| 3,4-Dihydroxybenzoic acid | 0.3 | 1 | 1 | 1 | 1 | 1 |
| eleutheroside E | 0.8 | 1 | 1 | 1 | 1 | 1 |
| hyperoside | 0.8 | 1 | 1 | 1 | 1 | 1 |
| isofraxidin | 0.6 | 1 | 1 | 1 | 1 | 1 |
| chlorogenic acid | 0.7 | 1 | 1 | 1 | 1 | 1 |

**Table S12.** Results of AHP analysis

| Component | Eigenvector | Weight value (%) | Maximum eigenvalue | Consistency Index (CI) | Consistency Ratio (CR) |
| --- | --- | --- | --- | --- | --- |
| syringin | 1.51 | 25.172 | 6.096 | 0.019 | 0.015 |
| 3,4-Dihydroxybenzoic acid | 0.817 | 13.616 |  |  |  |
| eleutheroside E | 0.936 | 15.601 |  |  |  |
| hyperoside | 0.936 | 15.601 |  |  |  |
| isofraxidin | 0.888 | 14.807 |  |  |  |
| chlorogenic acid | 0.912 | 15.204 |  |  |  |

**Figure S1**. Optimal classification methods for environmental variables


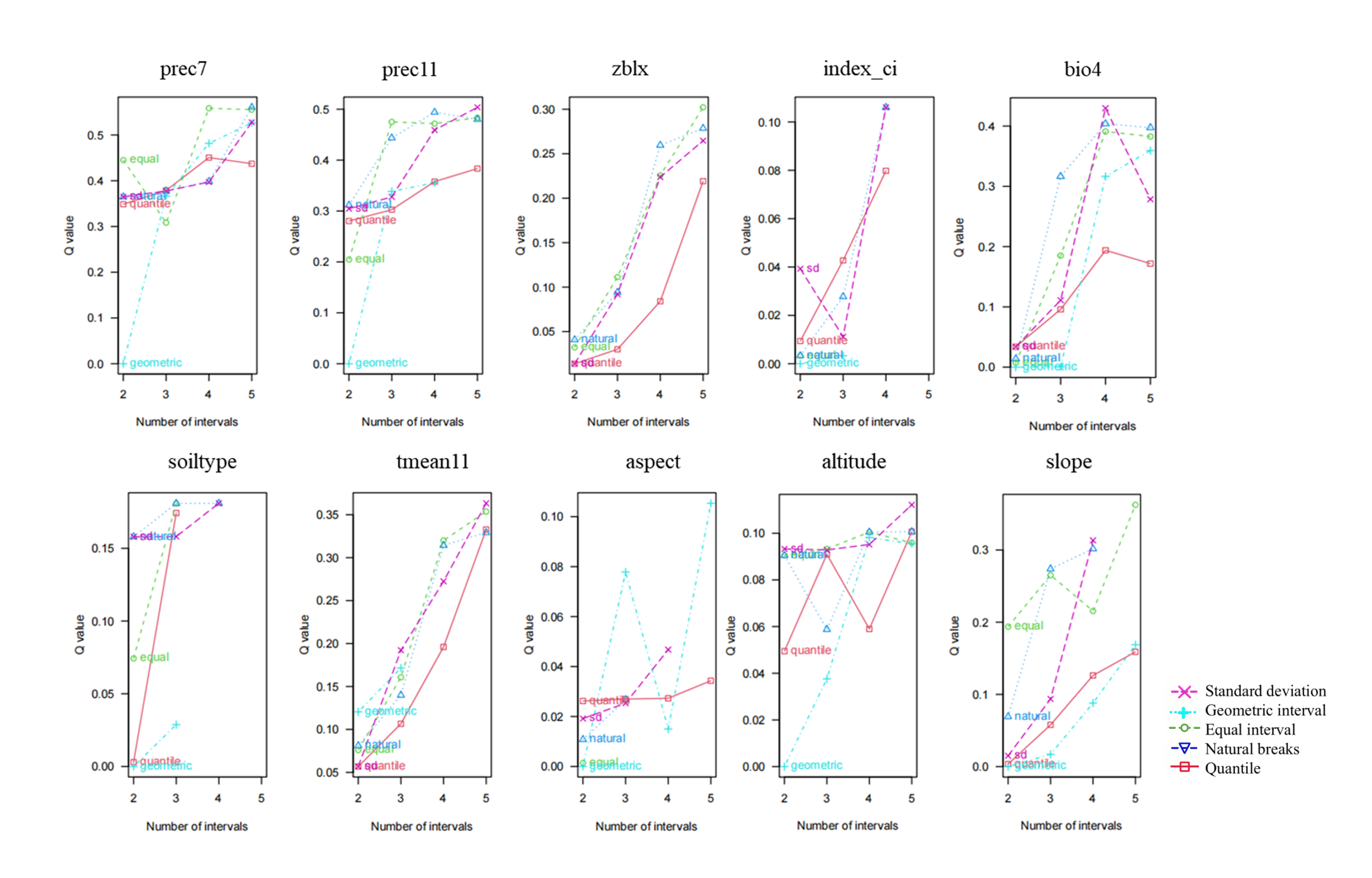


**Figure S2**. Optimal number of classes for environmental variable layers


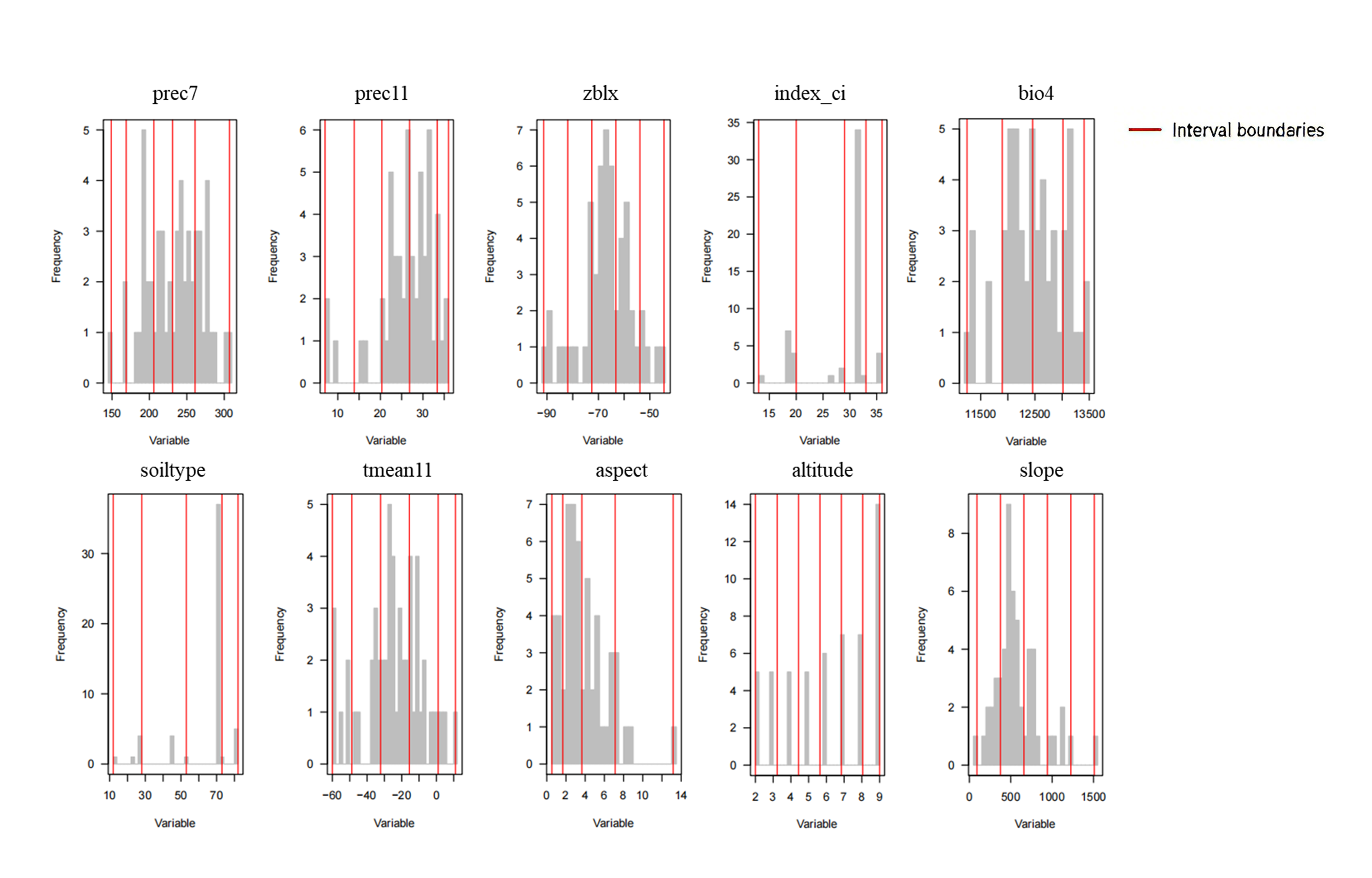


**Figure S3**. Results of factor detector

showing the significance and explanatory power (q-values) of environmental variables.


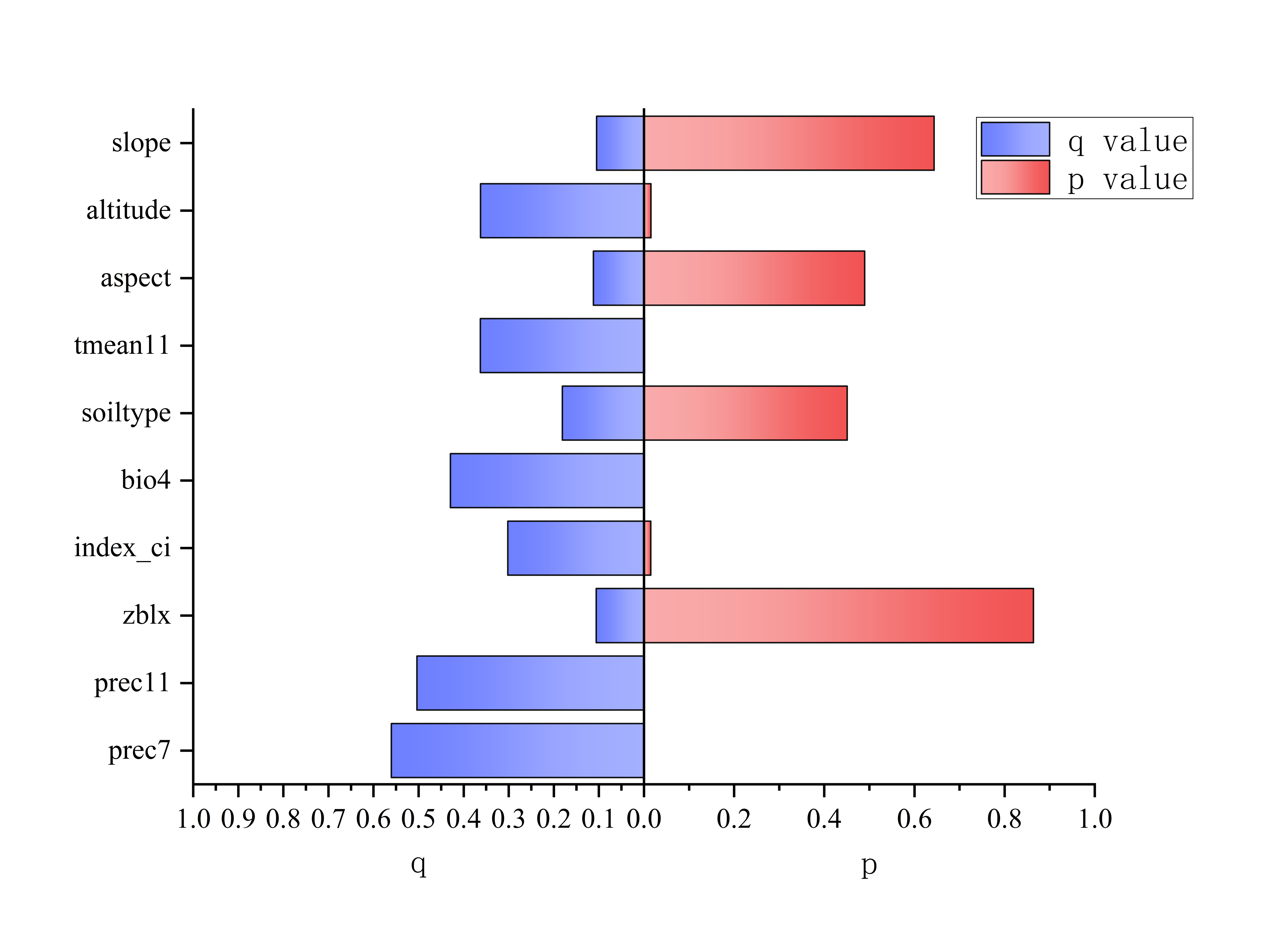


**Figure S4**. Results of interaction detector

showing the combined effects between pairs of environmental variables.


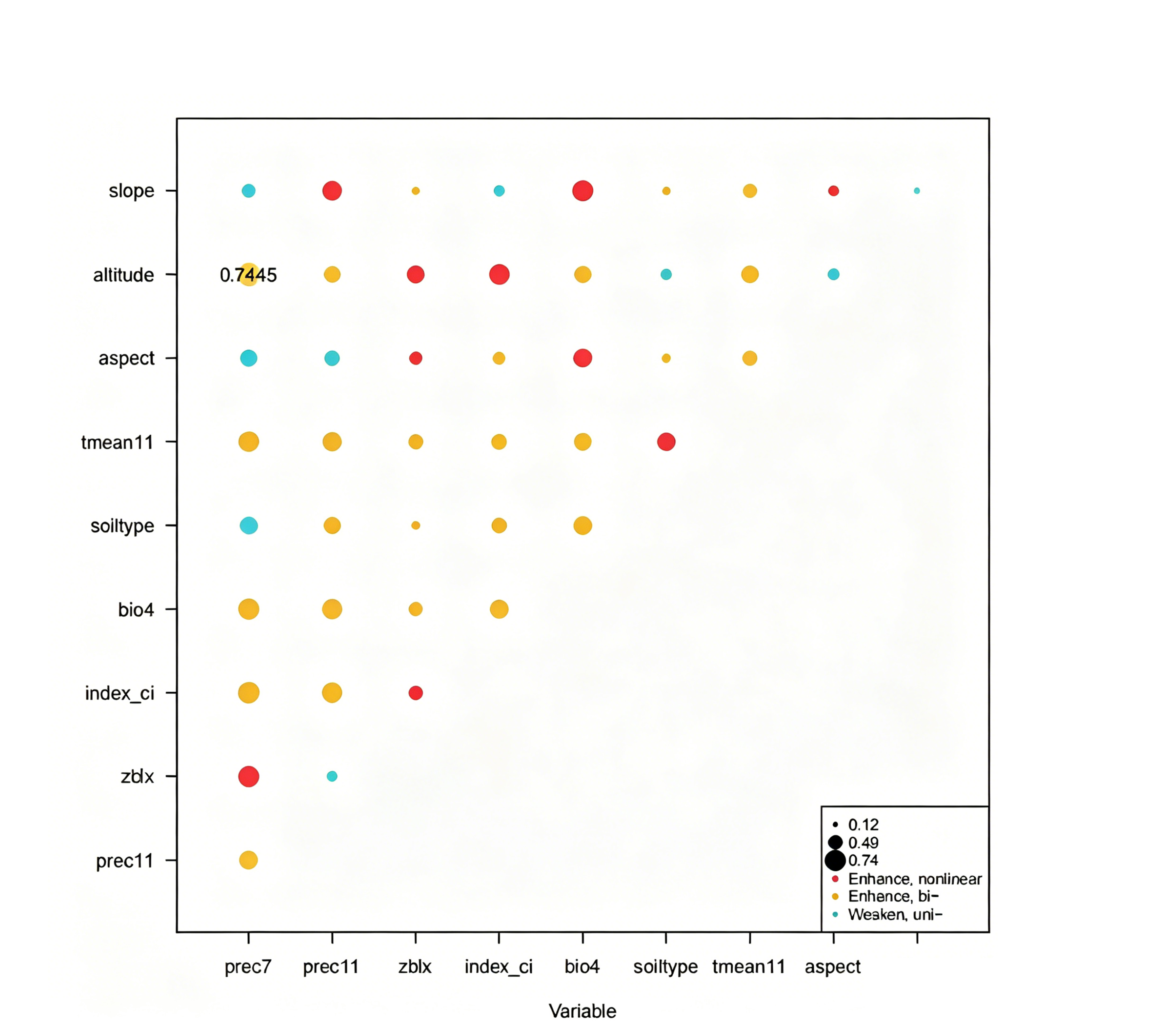


**Figure S4**. results of ecological detector

showing the comparison of significance between pairs of environmental variables.


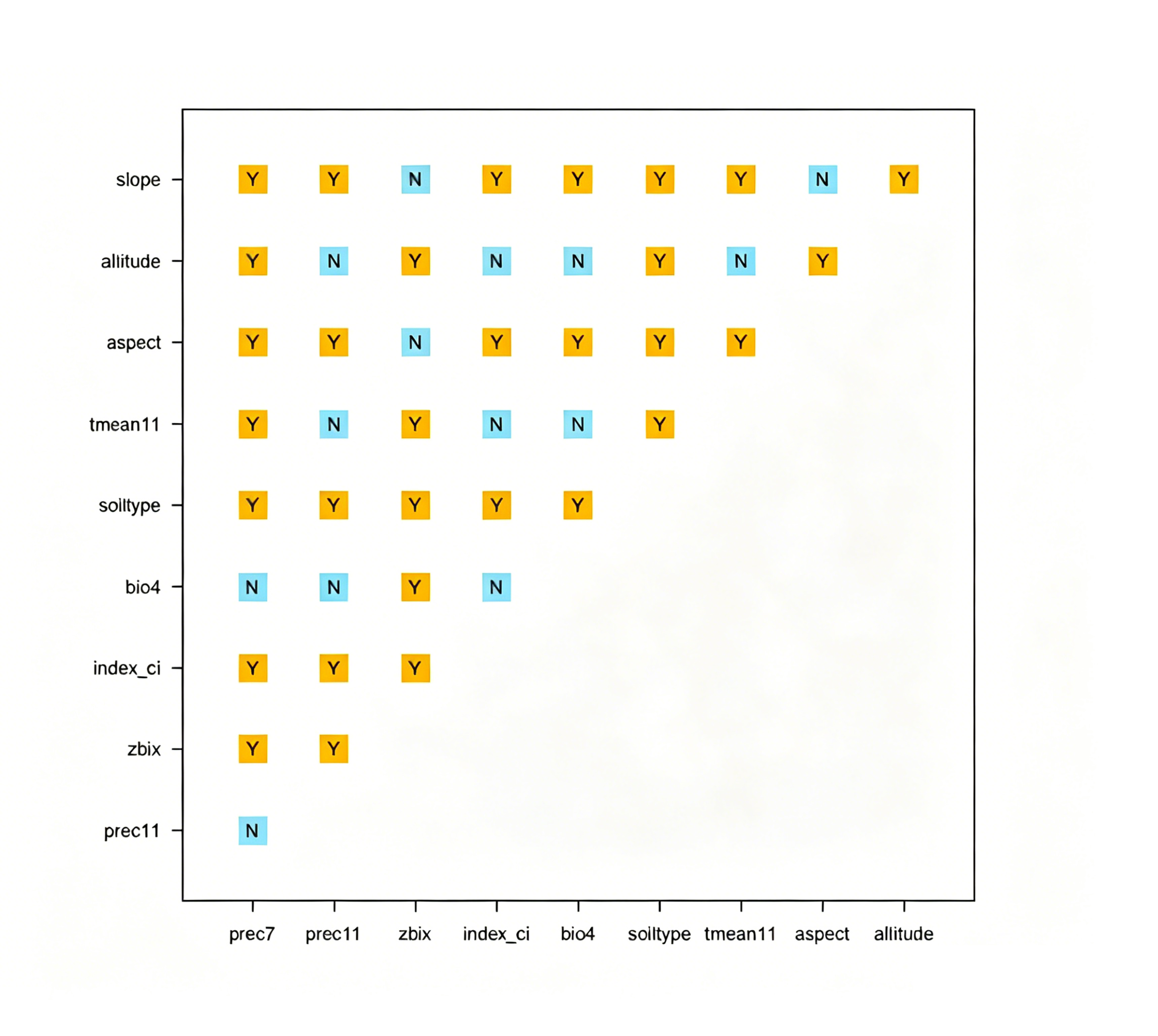


**Figure S6**. results of risk detector

showing whether the internal zones of each environmental variable are significantly different.


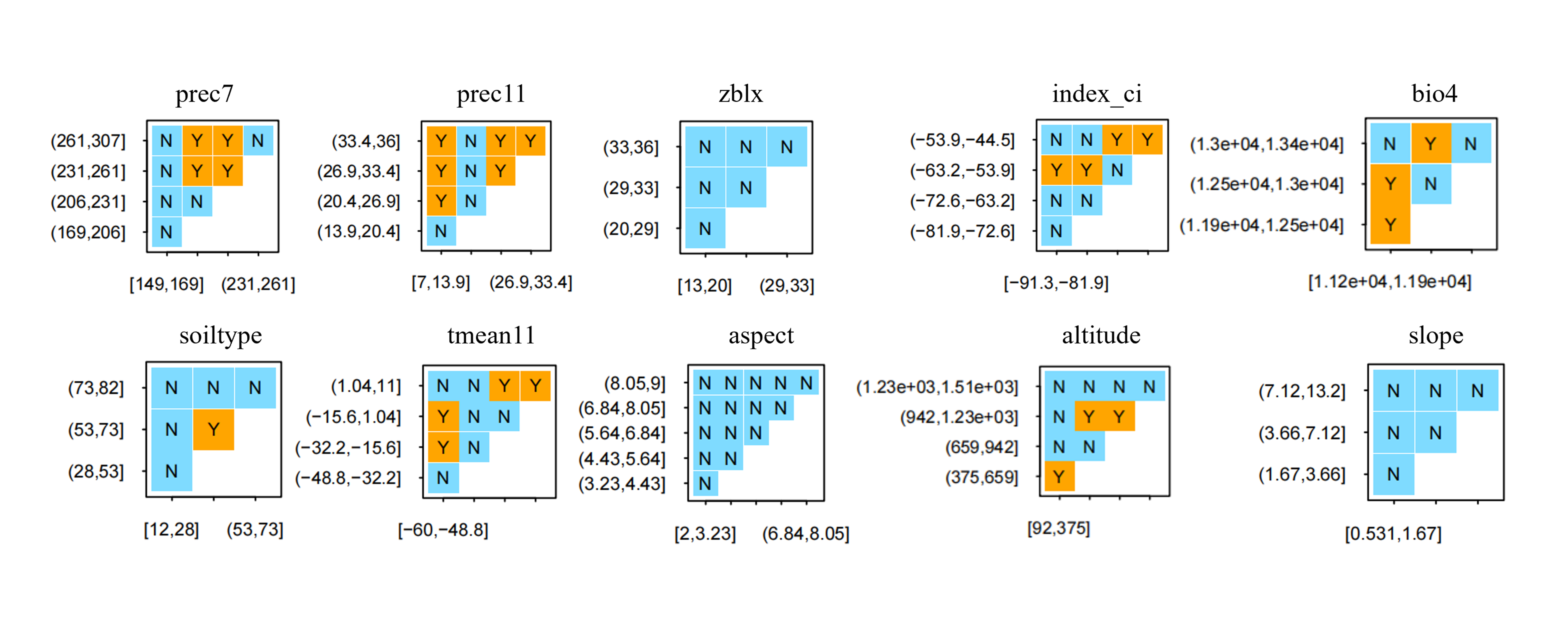

Supplement: Supplementary file 1 [file SupplementaryFile1.docx]
